# Multilevel Gene Expression Changes in Lineages Containing Adaptive Copy Number Variants

**DOI:** 10.1093/molbev/msaf005

**Published:** 2025-01-23

**Authors:** Pieter Spealman, Carolina de Santana, Titir De, David Gresham

**Affiliations:** Center for Genomics and Systems Biology, Department of Biology—New York University, New York, NY, USA; Laboratório de Microbiologia Ambiental e Saúde Pública—Universidade Estadual de Feira de Santana (UEFS), Bahia, Brazil; Center for Genomics and Systems Biology, Department of Biology—New York University, New York, NY, USA; Center for Genomics and Systems Biology, Department of Biology—New York University, New York, NY, USA

**Keywords:** gene expresssion, copy number variation, chemostat, adaptation, Ssd1, uORF

## Abstract

Copy number variants (CNVs) are an important class of genetic variation that can mediate rapid adaptive evolution. Whereas, CNVs can increase the relative fitness of the organism, they can also incur a cost due to the associated increased gene expression and repetitive DNA. We previously evolved populations of *Saccharomyces cerevisiae* over hundreds of generations in glutamine-limited (Gln-) chemostats and observed the recurrent evolution of CNVs at the *GAP1* locus. To understand the role that gene expression plays in adaptation, both in relation to the adaptation of the organism to the selective condition and as a consequence of the CNV, we measured the transcriptome, translatome, and proteome of 4 strains of evolved yeast, each with a unique CNV, and their ancestor in Gln- chemostats. We find CNV-amplified genes correlate with higher mRNA abundance; however, this effect is reduced at the level of the proteome, consistent with post-transcriptional dosage compensation. By normalizing each level of gene expression by the abundance of the preceding step we were able to identify widespread differences in the efficiency of each level of gene expression. Genes with significantly different translational efficiency were enriched for potential regulatory mechanisms including either upstream open reading frames, RNA-binding sites for Ssd1, or both. Genes with lower protein expression efficiency were enriched for genes encoding proteins in protein complexes. Taken together, our study reveals widespread changes in gene expression at multiple regulatory levels in lineages containing adaptive CNVs highlighting the diverse ways in which genome evolution shapes gene expression.

## Introduction

Copy number variants (CNVs) are amplifications or deletions of DNA that can span dozens of nucleotides to whole chromosomes. CNVs are frequently observed over both short and long evolutionary time spans, although their selective advantage may be different between the two (Kuzmin et al. 2022). In the short-term, CNVs can result in large changes in gene expression and protein abundance, which provides a selective advantage further driving rapid adaptive evolution (Kondrashov 2012; Myhre et al. 2013; Robinson et al. 2023). Gene amplification has been shown to mediate rapid adaptation to a variety of selective pressures from nutrient limitation to antibiotics in both natural and experimental populations of microbes (Gresham et al. 2008; Nair et al. 2008; Selmecki et al. 2009; Paulander et al. 2010; Pränting and Andersson 2011; Hong and Gresham 2014; Dhami et al. 2016; Hull et al. 2017; Lauer et al. 2018; Todd and Selmecki 2020). CNVs are also common in cancers, where they can promote tumorigenesis by oncogene amplification (Ben-David and Amon 2020) and drive genome-wide changes in gene expression (Shao et al. 2019).

A simple model of the short-term fitness effects of CNVs is a variant of the “driver-hitchhiker” model (Smith and Haigh 1974; Golzio and Katsanis 2013) wherein the fitness benefit of a CNV is derived from the amplification of a single gene or “driver,” whereas fitness costs arise from the amplification of “hitchhikers.” Since CNVs can include up to hundreds of genes, the potential fitness costs of hitchhikers can be high (Makanae et al. 2013; Avecilla et al. 2023). These fitness costs can be categorized as “dosage burden” wherein the fitness cost arises from the burden of the additional DNA replication and gene expression (Dekel and Alon 2005; Makanae et al. 2013; Bonney et al. 2015). Conversely, fitness costs may arise from the stoichiometric imbalance of specific “dosage sensitive” genes. The archetypal example of dosage sensitivity is the imbalance of proteins involved in a heteromeric protein complex, leading to negative impacts on complex formation and function (Wagner 2005; Veitia et al. 2008; Rice and McLysaght 2017). Importantly, the fitness effect of any CNV is highly dependent on the genetic background and environmental context (Sunshine et al. 2015; Robinson et al. 2023).

Dosage compensation (DC) is one mechanism by which an organism could mitigate the fitness costs of hitchhiker gene amplification. While classically associated with sex-chromosome inactivation through chromatin silencing (Straub and Becker 2007; Jordan et al. 2019), DC can also be gene specific (Straub and Becker 2007). Furthermore, while DC is often conceptualized as “complete,” such that additional gene copies result in no additional expression, DC can also result in a range of increased gene expression levels that are significantly less than what would be expected based on the copy number (Itoh et al. 2007; Brockdorff and Turner 2015). This “incomplete” DC is sometimes referred to as attenuation (Ascencio et al. 2021). Here, we use the term DC to refer to both “complete” (e.g. the gene expression from 2 copies of the gene is identical to the gene expression from 1 copy) and “incomplete” (e.g. the gene expression from 2 copies of the gene is greater than the gene expression from 1 copy, but <2 times the expression of 1 copy).

Recent work on the transcriptional regulation of CNV-amplified genes has found that, depending on genetic background and environment 10% to 60% of amplified genes show evidence of transcriptional DC (Springer et al. 2010; Gasch et al. 2016; Avecilla et al. 2023). The mechanism underlying transcriptional DC is unclear but changes in transcription factor abundances and their concentrations is one possible mechanism (Prestel et al. 2010). Alternatively, post-transcriptional mechanisms that impact mRNA stability may contribute to transcriptional DC.

Translational DC has recently become a focus of interest with the advent of ribosome profiling (Ingolia et al. 2009; Zhang and Presgraves 2017) but has also been studied using other methods (Gebauer et al. 1999; Krasovec et al. 2023). At the translational level, some global mechanisms have been proposed including spliceosomal or ribosomal components acting to limit total global expression in an organism (Veitia et al. 2013), and co-translational mechanisms, such as the unfolded protein response (Kane et al. 2021), although the universality of these mechanisms remains unresolved (Larrimore et al. 2020). Gene-specific mechanisms have also been proposed and identified, such as sequence specific RNA-binding proteins (RBPs) acting on specific genes and altering their post-transcriptional and translational regulation (Mayr 2017; Schultz et al. 2020). The archetypal case of translational DC is sex-chromosome DC in *Drosophila melanogaster*, enacted by binding of the RBP sex-lethal (SXL), which controls translation efficiency of *msl-2* mRNA through an upstream open reading frame (uORF) (Medenbach et al. 2011).

uORFs are short ORFs (6 to 300 nucleotides long) that are positioned upstream, in the transcript leader (TL, or 5′ UTR) of the main ORF (mORF) of a gene. uORFs have either canonical start codons (e.g. AUG) or near-cognate codon (Zitomer et al. 1984) translation initiation sites that allow for alternative translation initiation separate from the mORF, and as such can operate as translational regulatory elements, either preventing or enabling the translation of the downstream mORF (Hinnebusch 2005). While uORFs have been suggested to be a broad class of stress responsive regulatory elements (Lawless et al. 2009), their role in the CNVs has not previously been evaluated.

One interesting candidate for gene-specific translational regulation in *Saccharomyces cerevisiae* is *SSD1*, which encodes an RBP found to preferentially bind to cis-elements in TLs (Bayne et al. 2022) and alter translation (Hogan et al. 2008; Ohyama et al. 2010). SSD1 is believed to play an important role in the response of yeast to aneuploid stress (Hose et al. 2015, 2020). Whereas, initial research suggested that yeast exhibit high sensitivity to aneuploidy, subsequent research has shown that this sensitivity is strain dependent, and due to the loss of *SSD1* in the lab strain W303 (Hose et al. 2015, 2020). Whether *SSD1* has a role in accommodating CNVs in the genome is unknown.

Quantitative mass spectrometry has enabled the identification of DC at the level of protein abundance (Stingele et al. 2012; Senger et al. 2022). At the protein level, studies in yeast have suggested that 10% to 20% of gene amplifications exhibit significant protein abundance DC (Dephoure et al. 2014; Ishikawa et al. 2017), although genetic differences are a known source of variance (Hose et al. 2020; Larrimore et al. 2020). One proposed mechanism for DC at the protein level is that excess subunits of proteins associated with heteromeric complexes are selectively targeted for protein aggregation (Samant et al. 2019) and degradation by ubiquitination (Ishikawa et al. 2020; Senger et al. 2022). A recent study using hundreds of natural isolates reported that nearly all aneuploid yeast strains studied had consistent protein level DC with an average decrease of 25% from the expected abundance (Muenzner et al. 2024).

Here, we undertook an analysis of multilevel gene expression changes in long-term experimentally evolved lineages of *S. cerevisiae* with acquired adaptive CNVs. These strains evolved over the course of hundreds of generations in glutamine-limited chemostats and contain distinct adaptive CNVs containing the same driver gene *GAP1* (Lauer et al. 2018) but distinct sets of additional hitchhiker genes within the CNV, numbering between 18 and 82. We quantified expression at the transcriptional, translational, and protein levels in glutamine-limited chemostats. We found over 2,000 genes were significantly different in transcript abundances and ribosome occupancy, and over 1,700 genes were significantly different in protein abundance.

By normalizing each sequential step of gene expression to account for changes at the preceding level, we were able to identify changes in gene regulation that alter the efficiency, including both increases and decreases, of expression at each level. Across the entire genome, we find evidence of widespread changes in gene expression efficiency at the transcriptional (median 25%), translational (median 11%), and protein (median 8.5%) levels. Relative to these genome-wide changes, we find that more CNV-amplified genes have lower efficiencies at every level of expression. However, it is only at the level of protein expression efficiency this is significantly different from the global rate.

We find that many genes with significant differences in expression efficiency are also enriched for potential regulatory mechanisms. Genes with significantly different translation efficiencies are enriched in both uORFs and Ssd1 binding site motifs, as well as the co-occurrence of uORFs and Ssd1 binding sites. Genes with significantly different protein expression efficiency are also significantly enriched in genes associated with protein complexes, suggesting stoichiometric imbalances may lead to targeted protein degradation.

Notably, each mechanism that we identify as contributing to DC is also involved in the regulation of gene expression more broadly and is not specific to CNV-amplified genes. This indicates that DC mechanisms in yeast that act on additional copies of genes are not unique mechanisms but rather, extensions of the suite of gene regulatory mechanisms that act to maintain robust phenotypes in response to diverse cellular and environmental stresses.

## Results

### Topological Structure of CNV Alleles

**Fig. 1.**
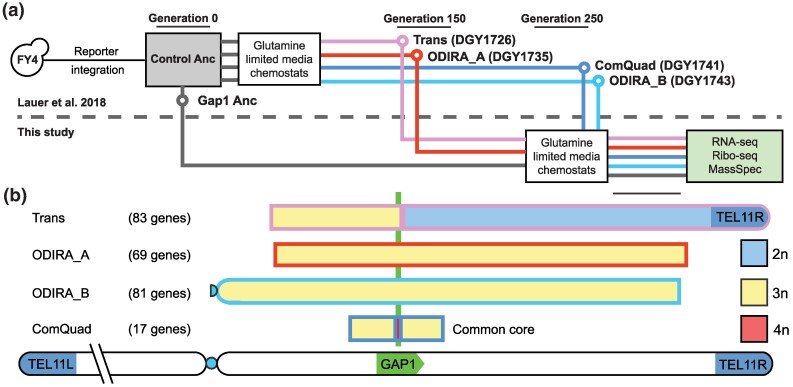
Study design and characterization of CNV strains. a) Strain provenance (Lauer et al. 2018; above the dotted line) and experimental design to assess gene expression effects using RNA-seq, ribosome profiling (Ribo-seq), and TMT-labeled mass spectrometry (this study, beneath dotted line) in glutamine-limited chemostats. b) A schematic showing the *GAP1*-locus amplified genes in each strain and their copy number. A common core of 17 genes is amplified in every evolved strain.

We analyzed 4 lineages that contained CNVs at the *GAP1* locus following long-term selection in glutamine-limited chemostats. CNV-containing lineages were identified through the use of a CNV reporter system (Lauer et al. 2018; Spealman et al. 2023) and isolated using FACS from heterogeneous populations following more than 150 generations of selection in glutamine-limited chemostats (Fig. 1a). Because defining the gene expression consequences of copy number (CN) variation requires a precise identification of the CN of each gene we performed hybrid de novo genome assembly (Spealman et al. 2022), using a combination of long-read (Oxford Nanopore Technologies) and short-read (Illumina) sequencing (Fig. 1b) with manual refinement of regions with large DNA secondary structures (Spealman et al. 2020). On the basis of the CNV breakpoints, we also inferred the likely mechanisms of formation for each CNV, which include transposon mediated tandem amplification and origin of replication dependent amplification (ODIRA; Brewer et al. 2011). The 4 unique CNV alleles containing *GAP1* were named to reflect these mechanisms.

CNV structures include “Trans,” a transposon mediated tandem duplication, spanning the genes from *TOF2* to *GAP1*, nested within a translocation and telomere replacement on ChrVIII (supplementary fig. S1c, Supplementary Material online). The Trans strain also contains a second CNV spanning *SAK1* to *YER137C* on chromosome V (supplementary fig. S2, Supplementary Material online). “ODIRA_A” is an ODIRA triplication spanning 77 genes (bounded by the genes *ALY1*-*SIR1*) (supplementary fig. S1d, Supplementary Material online) and “ODIRA_B” is an ODIRA triplication spanning 91 genes (bounded by the genes *VPS1*-*ESL2*) (supplementary fig. S1f, Supplementary Material online). Finally, “ComQuad” is a complex quadruplex that spans the common core of 17 genes from *SET3* to-*TRK2* resulting from an ODIRA driven triplication followed by a singular amplification of *GAP1* mediated by a transposon event (supplementary fig. SF1e, Supplementary Material online). Using methods previously described (Spealman et al. 2023) we unambiguously determined the CN of each gene in each CNV (supplementary table S1, Supplementary Material online) and also identified and filtered those genes that were disrupted by CNVs (**S2-S6**) resulting in a total of 107 CNV-amplified genes. Each of the 4 CNV strains included a subset of these 107 genes present at 2, 3, or 4 additional copies. A set of 17 amplified genes centered on the *GAP1* gene is common to all strains (the “common core”). A median of 2 de novo SNVs or indels were also identified per strain (supplementary fig. S7, Supplementary Material online). Importantly, none of the SNVs or indels occurs within genes amplified by CNVs, ensuring that gene CN is the only variation at these loci. Notably, the identical sequence of all amplified gene copies precludes identification of allelic differences in expression.

To assess the gene expression consequences of both the CNV allele and adaptation to nutrient limitation, we analyzed the 4 evolved CNV strains and the ancestral wildtype in glutamine-limited chemostats. Strains were grown until reaching a steady-state condition during which culture density remained constant (Gresham and Dunham 2014). All RNA-seq and ribosome profiling experiments were performed in tandem using 2 biological replicates for each strain. Mass spectrometry was conducted using 5 biological replicates to avoid high false detection rates (Martinez-Val et al. 2016; Brenes et al. 2019). Correlation between replicates was high (median rho = 0.99, supplementary table S2, Supplementary Material online) as were correlations between strains (median rho = 0.95, supplementary table S3, Supplementary Material online).

### Divergence of Gene Expression at Multiple Levels in Evolved Strains

**Fig. 2.**
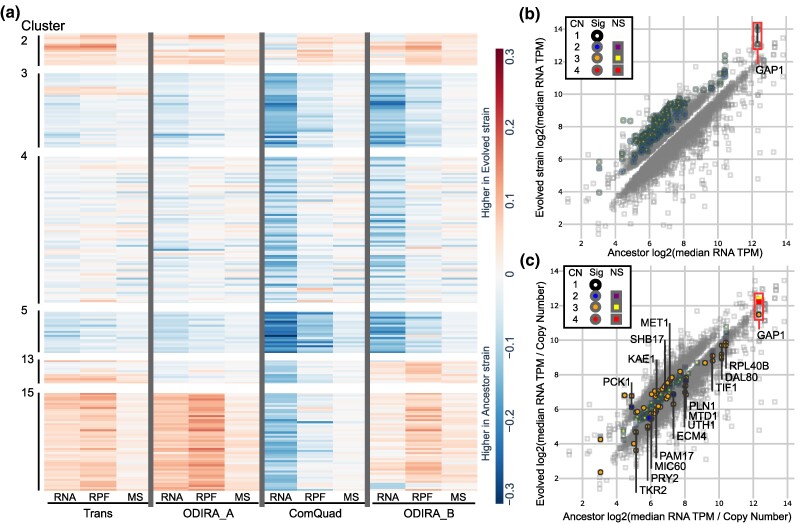
Gene expression divergence in evolved lineages containing CNVs. a) A clustered heatmap of the ratio of evolved over ancestral transformed data (methods) expression at multiple levels (RNA, RPF, and MS) of genes from all strains. The 218 genes shown are all significantly different in abundance (DESeq2 (RNA, RPF); Welch's *t*-test (MS); BH adj. *P*-value < 0.01) at multiple levels of gene expression, or are CNV amplified in at least 1 strain. b) Scatterplot of RNA abundance of genes with significantly different (DESeq2, BH adj. *P*-value < 0.05) expression between each of the 4 evolved strains and the ancestor. All CNV-amplified genes are significantly higher in the evolved strain. c) To assess if there were changes in transcription efficiency, we corrected RNA abundance for gene CN and found that a minority of genes in each strain (median of 36%) exhibit significantly different CN-corrected expression (DESeq2, BH adj. *P*-value < 0.05).

We first sought to identify genome-wide differences in gene expression between each evolved strain and the ancestor. We retained 4,289 genes after filtering genes poorly represented in any strain at any level of expression. Of the 107 CNV-amplified genes, 90 passed this QC filtering. We found 2,452 genes were significantly different in RNA abundance in at least one evolved strain (DESeq2, Benjamini–Hochberg (BH) adjusted *P*-value < 0.05, supplementary table S4, S8f, Supplementary Material online). Similarly, 2,181 genes were significantly different in ribosome protected fragment (RPF) abundance (DESeq2, BH adj. *P*-value < 0.05, supplementary table S5, S8g, Supplementary Material online). Finally, 1,784 genes were significantly different in MS abundance (Welch's *t*-test, BH adj. *P*-value < 0.05, supplementary table S6, S8h, Supplementary Material online). As the scale and nature (i.e. discrete vs. continuous) of the 3 different datatypes differ, we transformed the data to enable direct comparison between assays (Methods). To visualize gene expression across multiple levels we determined the difference in expression between each evolved CNV strain and the ancestor strain (Fig. 2a;  supplementary fig. S9, Supplementary Material online). We used k-means clustering on all levels of expression across all evolved strains (supplementary table S7, Supplementary Material online) and found some clusters are significantly enriched in genes associated with specific gene ontology (GO) terms (Saccharomyces Genome Database, BH adj. *P*-value < 0.05). For example, clusters 3, 4, and 5, which exhibit lower in expression in the evolved strains, are significantly enriched in genes associated with the following functions: cluster 3 is enriched in “protein catabolic process” (GO:0030163); cluster 4 with “protein-containing complex organization” (GO:0043933); and cluster 5 with “energy derivation by oxidation of organic compounds” (GO:0015980). Notably, clusters 2, 13, and 15, which tend to be higher in expression in the evolved strains, were not enriched in any GO terms; however, they were significantly enriched in genes amplified in CNVs (Fisher's exact test (FET), *P*-value < 0.05). Indeed, over 47 of the 57 genes in cluster 15 are genes amplified in CNVs in trans, ODIRA_A, and ODIRA_B. This set of genes comprises the genes outside of the common core of 17 genes, as can be seen with the lower relative expression of these unamplified genes in ComQuad.

### Differences in Transcription Efficiency in CNV-Amplified Genes

All CNV-amplified genes were found to have higher RNA abundance than the ancestor including the putative driver gene *GAP1*, which is one of the most highly expressed genes in this condition (Fig. 2b). Typically, in differential expression analysis the copy number of the underlying genes are assumed to be uniform throughout the genome and between genotypes. As that is not the case in our study, we first sought to correct for changes in copy number. Here, we define a null model in which the expected RNA abundance for genes within a CNV is determined by the abundance in the ancestor multiplied by the copy number in the evolved strain. For example, a 2-fold increase in gene copy number is expected to result in a 2-fold increase in mRNA. We determined the CN-corrected gene expression for all genes and then compared the observed RNA abundance to this expected value to quantify differences in transcription efficiency (Fig. 2c;  supplementary table S8, Supplementary Material online).

Previous work has described gene-specific transcription DC of CNV-amplified genes (Straub and Becker 2007; Ascencio et al. 2021). We find that almost half (43 of 90) of the CNV-amplified genes have significantly different RNA abundances from what would be expected given their copy number. We found significantly increased transcription efficiency for 22 CNV-amplified genes, with 4 genes (*PCK1*, *KAE1*, *SHB17*, and *MET1*) being significantly higher in multiple lineages, suggesting increased transcription efficiency. Conversely, we found significantly lower RNA transcription efficiency in 24 genes, with 12 genes showing decreased transcription efficiency in all strains (*TKR2*, *PRY2*, *MIC60*, *PAM17*, *ECM4*, *UTH1*, *MTD1*, *PLN1*, *TIF1*, *DAL80*, *RPL40B*, and *GAP1*). Interestingly, we find that *GAP1*, the hypothesized driver of CNV selection, has lower transcription efficiency in all evolved strains, although this reduction is only significant in 2 of the CNV strains (Trans and ODIRA_A). *DAL80*, a transcription factor that is the negative regulator of *GAP1* (Daugherty et al. 1993) and located <10 kb from *GAP1* on chromosome XI and is within the common core, has significantly lower transcription efficiency in all evolved strains.

Across all strains, we find a median of 19% of CNV-amplified genes per strain have significantly lower transcription efficiency. While this supports the model of transcription efficiency as a mechanism of DC, decreased transcription efficiency is not limited to amplified genes. Genome wide, we find a median of 678 unamplified genes per strain (16%) also have significantly decreased transcription efficiency. While amplified genes are 1.38-fold enriched in genes with lower transcription efficiencies, this is not significantly different for the global background (FET, median *P*-value = 0.35). No significant trend was observed for genes with increased transcription efficiency (supplementary table S9, Supplementary Material online). This suggests that the decreased transcription efficiency observed in CNVs may not be produced by a mechanism unique to them.

### Changes in Translation Efficiency

To investigate how changes in translational regulation may act to alter gene expression, we normalized the ribosome abundance, as measured by RPFs aligned to mORF protein coding sequences only, by transcript abundance (supplementary table S10, Supplementary Material online). This translation efficiency ratio is an estimate of the amount of translating ribosomes per transcript (Ingolia et al. 2009) and can be the result of altered translation initiation rates at the start codon as well as differences in elongation rates throughout the mORF (Weinberg et al. 2016).

In total, 21 CNV-amplified genes were found to have significantly different translation efficiencies (FET, *P*-value < 0.05). Of these, 9 had significantly higher translation efficiency in the evolved strain relative to the ancestor with 5 genes (*PLN1*, *PAM17*, *MTD1*, *RPL40B*, and *DID2*) being higher in multiple backgrounds. Conversely, 9 genes had significantly lower translation efficiency relative to the ancestor but only 2 were significantly lower in multiple backgrounds: *TIF1* in both Trans and ODIRA_A, as well as *GAP1* in every background (supplementary table S10, Supplementary Material online). Finally, 3 genes were differentially expressed in specific strains, *DAL80* was higher in Trans and lower in ODIRA_B; *RPS21A* was higher is ODIRA_A but lower in both Trans and ODIRA_B; while *UTH1* was lower in ODIRA_A and higher in both ODIRA_B and ComQuad.

**Fig. 3.**
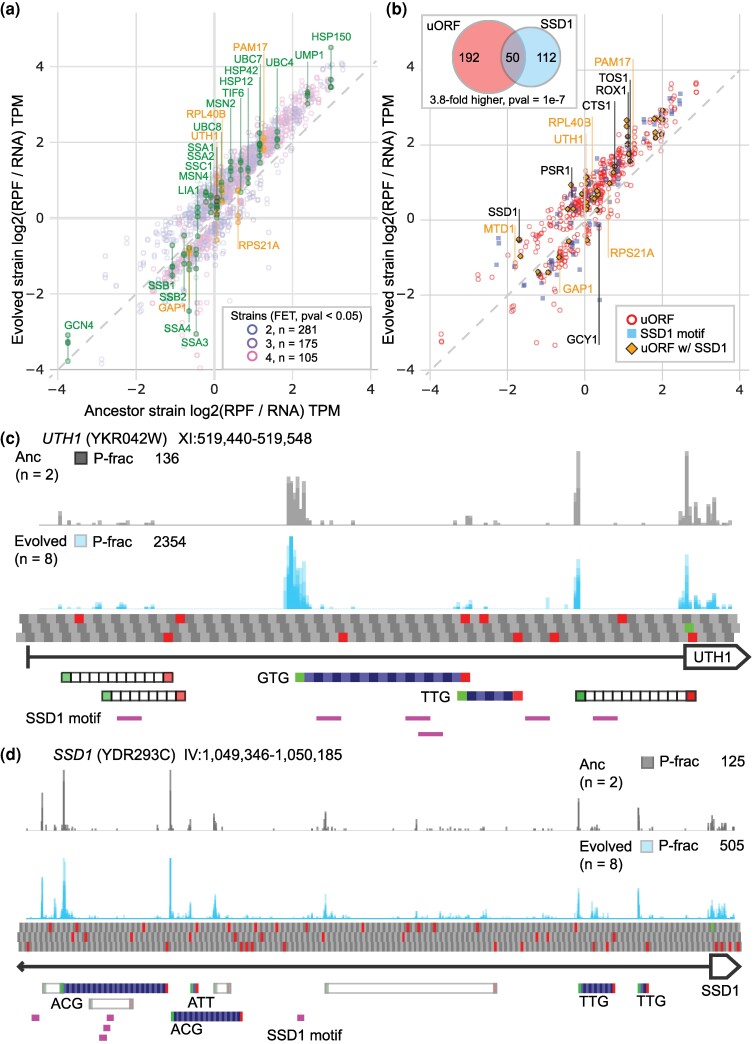
Divergence in translation efficiencies and potential regulatory mechanisms. a) 561 genes have significantly different translation efficiency relative to the ancestor in at least 2 evolved strains (FET, *P*-value < 0.05). Gene names in orange indicate CNV-amplified genes. b) Genes with significantly different translation efficiency and the presence of a high confidence uORF (circle), significant SSD1 motifs (square), or both (3.8-fold higher than expected at random, HGM, *P*-value = 1e−7). c) Ribosome binding within the TL (line) of *UTH1* (box) in the ancestor and evolved strains. High confidence predicted uORFs are shown in gray, start codons and stop codons, low confidence uORFs are hollow boxes. Ssd1 binding motifs are shown in purple. The 5′ terminus of the transcript is denoted by the flat arrowhead. d). Example of uORFs and *SSD1* binding motifs upstream of *SSD1*. The 5′ terminus of the transcript extends between the indicated region as indicated by the pointed arrowhead.

For the unamplified genes, we found that 1,011 genes had significantly different translation efficiencies relative to the ancestor across all 4 strains (Fig. 3a, FET, *P*-value < 0.05), with a median of 121 genes per strain. The majority (655, 65%) had significantly higher translation efficiency than the ancestor, with 371 genes being higher in multiple backgrounds, suggesting they are translationally upregulated. Conversely, only 279 (28%) of these had significantly lower translation efficiency than the ancestor, with 100 being lower in multiple backgrounds. Finally, 77 genes (8%) were significantly different in opposite directions in specific strains.

Across strains, we find that a median of 5% of CNV-amplified genes have significantly lower translational efficiencies, which is not significantly higher (FET, median *P*-value = 0.32) than the observed median rate of 3% in unamplified genes. No significant trend was observed for genes with increased translation efficiency (supplementary table S10, Supplementary Material online).

### Inference of Potential Mechanisms of Translational Gene Regulation

Previously published research has identified potential uORFs upstream of several genes that are amplified by CNVs in one or more of our strains, including *IRS4*, *ALY1*, *NTR2*, *KAE1*, *UIP5*, *TFA2*, *TGL4*, and *MLP1* (May et al. 2023). We developed uORFish, a uORF identification tool that uses ribosome profiling features on molecularly validated uORFs (supplementary fig. S10, Supplementary Material online) to train a machine learning model to analyze ribosome profiling data from each strain (Methods, supplementary fig. S11, Supplementary Material online). We identified 875 genes with high confidence uORFs genome wide, 706 of which remain after filtering low quality genes, of these 242 exhibit significantly different translation efficiency in one or more strains (Fig. 3b), which is significantly higher than expected at random (1.4-fold higher, hypergeometric method (HGM) *P*-value = 6.3e−12). As uORFs are often associated with lower translation efficiencies, it may be the case that CNV-amplified genes with uORFs may more often be translationally downregulated. However, although genes that are both significantly lower in translational efficiencies and contain uORFs that are ∼3-fold enriched in CNVs (5 out of 12) this rate is not significantly different (FET, *P*-value = 0.079) compared to those not amplified by CNVs (74 out of 369).

Recently, Ssd1, an RBP, previously associated with aneuploidy tolerance (Hose et al. 2015, 2020) was identified as having targets specific to stress conditions using an analysis of high confidence binding sites in optimal and heat-shock conditions (Bayne et al. 2022). To investigate the role Ssd1 may play in gene regulation in evolved CNV strains, we evaluated genes significantly enriched in the conserved “CNYUCNYU” motif. Using the CNYUCNYU motif, we scanned the annotated TLs of the genome to identify genes enriched in the Ssd1 binding motif, identifying 217 significantly enriched genes (FET, *P*-value < 0.05) of which 162 were present in our data set (Fig. 3b).

We found genes with uORFs were also enriched in Ssd1 binding motifs within their TLs (1.9-fold higher, HGM, *P*-value = 2.6e−06). We also found that genes with significantly different translation efficiencies were enriched in Ssd1 binding motifs (1.4-fold higher, HGM, *P*-value = 3.7e−3) and that genes with significantly different translation efficiency were 4.3-fold more likely to have both Ssd1 motifs and uORFs (HGM, *P*-value = 7.1e−06). Given the potential for interaction between uORFs and Ssd1 binding motifs in TLs, we used a relative genomic distance test co-occurrence (Favorov et al. 2012). We found that Ssd1 motifs and uORFs were co-occurrent at significantly higher rates than expected by chance over very close proximities (supplementary fig. S12, Supplementary Material online).

One well-known mRNA target of Ssd1 is *UTH1*, a mitochondrial protein involved in regulating both mitochondrial biogenesis and degradation (Camougrand et al. 2004). Overexpression of *UTH1* has been shown to lead to cell death in yeast strains lacking functional *SSD1* (Camougrand et al. 2003) and is associated with the role of Ssd1 in cellular response to aneuploid stress (Hose et al. 2020). Our analysis of the ribosome profiling data found a large abundance of uORFs and numerous CNYUCNYU motifs in *UTH1* (Fig. 3c). Similar co-occurrences of uORFs and CNYCNYU motifs were observed for several well described Ssd1 targets including *SUN4*, *CTS1*, *DSE2*, *SRL1*, and *SCW10* (Bayne et al. 2022) as well as *TOS1* (Hose et al. 2020). Other well-known targets of Ssd1 such as *CLN2* (Ohyama et al. 2010), *CIC1* (previously *NSA3*), *SCW4*, and *CCW12* (Bayne et al. 2022) also had evidence of uORFs but scored below the uORFish default quality threshold in one or more replicates. Importantly, we also found that *SSD1* itself appears to have extensive uORFs and Ssd1 motifs, suggesting it may autoregulate its own expression (Fig. 3d). Notably, while *SSD1* has significantly lower mRNA abundance (DESeq2, *P*-value < 0.05) in every evolved strain compared to the ancestor, it is translationally upregulated in all evolved strains, resulting in final protein abundance slightly higher than the ancestor, consistent with translational regulation.

### Differences in Protein Expression Efficiency

As with transcription abundance and ribosome occupancy, although many CNV-amplified genes have higher protein abundance relative to the ancestor, not all do, suggesting changes in protein expression efficiency (supplementary figs. S13 to S16, Supplementary Material online). Differences in protein expression efficiency (i.e. the ratio of protein to ribosome abundance) can be evidence of post-translational regulation, as is likely the result of changes in protein stability (Ishikawa et al. 2020; Senger et al. 2022). However, unlike the translation efficiency estimation for which both RNA and RPF abundance measurements are produced using comparable methodologies and produce units of similar scale; protein abundance, as measured by TMT-labeled mass spectrometry, and ribosome profiling are substantially different making direct ratio calculations challenging. To address this challenge, we use 2 approaches (each detailed in the “Material and Methods” section). As generalized linear models (GLMs) are feature scale agnostic, one method is to use a GLM on untransformed data to identify significant outliers, this is a powerful but overly conservative method (Methods, supplementary fig. S18, Supplementary Material online). In addition to this, we first transform both MS and RPF abundances to a similar numerical scale (Methods, supplementary fig. S17, Supplementary Material online) and then identify significant outliers in expression efficiency compared to similarly expressed genes (e.g. FDR 5%), (Methods, supplementary fig. S19, Supplementary Material online). The combination of these methods (here referred to as transformed near-neighbor test with GLM, or TNNTg) performs well on test data (Methods, supplementary fig. S20, Supplementary Material online) and was used to identify genes in CNV-containing strains with significant differences in protein expression efficiency relative to the ancestor (supplementary table S11, Supplementary Material online).

**Fig. 4.**
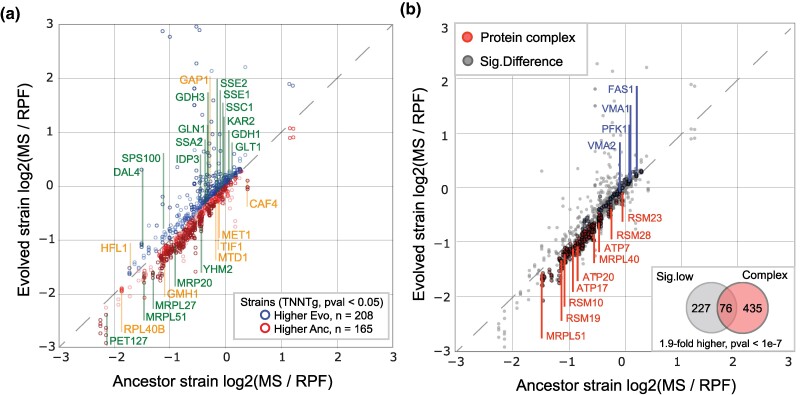
Divergence in protein expression efficiencies and potential regulatory mechanisms. Evolved and Ancestor strain protein expression efficiency ratios for a) genes with significantly different protein expression efficiency relative to the ancestor in at least 2 evolved strains) and for b) genes that have significantly different protein expression efficiency and are part of multimeric protein complexes are shown either above the diagonal (higher efficiency in Evolved strain) or below (lower efficiency in Evolved strain). Genes with significantly lower protein expression efficiency are 1.9-fold higher than random, HGM, *P*-value < 1e−7).

Using this approach, we find 624 unamplified genes and 46 amplified genes (Fig. 4a) with significantly different protein expression efficiencies in evolved lineages compared to the ancestor. Of the unamplified genes, the majority 454 genes (73%) have significantly higher efficiency with 353 being significantly higher in multiple backgrounds. These were significantly enriched in genes (*IDP3*, *GDH1*, *GDH3*, *GLN1*, and *GLT1*) associated with the glutamate biosynthesis pathway (intermine pathway PWY3O-13, HGM, BH adj. *P* = 3.91e−3) and genes associated with protein refolding (GO:0042026, HGM, BH adj. *P* = 1.86e−7) including members (*KAR2*, *SSA2*, *SSC1*, *SSE1*, and *SSE2*) of the HSP70 family (Yam et al. 2005). Conversely, 303 genes (48%) have lower efficiency, with 193 being significantly lower in multiple backgrounds. These were found to be enriched in genes associated with mitochondrial translation (45 genes, GO:0032543, HGM, BH adj. *P* = 5.60e−38) and oxidative phosphorylation (11 genes, GO:0006119, HGM BH adj. *P* = 1.11e−11). Finally, 96 genes (11%) were significantly different in opposite directions in specific strains.

We find the opposite trend with the CNV-amplified genes. A total of 45 CNV-amplified genes have significantly different protein expression efficiencies, with the majority of these (27 or 59%) being significantly lower than the ancestor with 12 being significantly lower in multiple strains. By contrast, 12 (26%) genes were significantly higher in protein expression efficiency with 5 being significantly higher in multiple strains (Fig. 4a, orange). Finally, 7 (15%) genes were significantly different in opposite directions in specific strains.

Notably, although small in number, CNVs are a median of 6.9-fold enriched in genes with significantly lower protein expression efficiency relative to the rest of the genome (FET, median *P*-value = 5.4e−3). Previous studies of DC at the protein level have found evidence of targeted degradation of proteins involved in heteromeric complexes (Ishikawa et al. 2017), potentially through the ubiquitination of the exposed interfaces of unpaired heteromers (Ishikawa et al. 2020). To test this, we looked for an enrichment of genes that encode proteins known to be involved in heterodimer complexes within the subset of genes with significantly lower protein expression efficiency. We found that genes with significantly lower protein expression efficiency were 1.9-fold enriched in proteins involved in heterodimer complexes (FET, *P*-value = 1e−7).

## Discussion

In this study, we set out to characterize how gene expression changes at the transcriptional, translational, and protein level in response to adaptive CNVs that are selected in response to strong selection. We quantified multiple aspects of gene expression in 5 strains of yeast, 4 of which had undergone adaptation to glutamine-limited chemostats for hundreds of generations and had gained CNVs, as well as an ancestral strain unadapted to glutamine-limited chemostats and lacking any CNVs (Fig. 1).

In a comparison of gene expression at the level of transcription and translation, we observed over 2,000 genes had significant changes in expression and over 1,700 genes had significant changes in protein abundance (Fig. 2a). The majority of genes amplified by CNVs had significantly higher levels of expression than their ancestral counterparts (Fig. 2b), which agrees with previous reports on the broad effects of CNVs (Springer et al. 2010; Gasch et al. 2016; Avecilla et al. 2023). We also observed that the number of significantly divergent genes (including both amplified and unamplified) decreases at each successive level of expression, from a median of ∼1,500 per strain for RNA, to ∼1,100 for ribosome abundance, to ∼600 per strain for protein abundance. This agrees with previous reports of buffering of divergent gene expression at the translational (McManus et al. 2014; Kusnadi et al. 2022) and post-translational levels (Wang et al. 2018; Jiang et al. 2023). Furthermore, we find that many of these genes exhibit significantly different expression in multiple lineages (supplementary tables S4 to S6, Supplementary Material online). These parallel changes of gene expression in strains with CNVs is consistent with potential physiological responses acting in response to the CNVs, in glutamine-limited growth conditions, to increase relative fitness by mitigating the fitness cost of CNVs.

Since the level of expression at each stage of gene expression is dependent on the abundance of the molecule that precedes it, we sought to identify changes in gene regulation that alter the efficiency of expression at each level by normalizing each step of gene expression by the preceding step. This enabled us to evaluate changes in gene expression efficiencies between the evolved strains and the ancestor (Figs. 2c, 3a, and 4a). By comparing the observed RNA abundance to what would be expected given the ancestral expression and the copy number (Fig. 2c), we found changes in transcription efficiency to be widespread impacting a median of 25% of all genes per strain. While CNVs had slightly higher rates of genes with either significantly higher or lower transcriptional efficiency, this effect was not significantly different from the global background.

We found a median of 11% of all genes per strain exhibited significant changes in translation efficiencies relative to the ancestor. While considerably less than the 25% of genes with significant differences in transcription efficiencies, this decrease could either be due to the more limited role of changes in translational efficiency or a difference in the statistical powers of the 2 tests used.

Finally, we found that genes with significantly different protein expression efficiency are rare, with only a median of 8.5% of all genes per strain. Given the conservative nature of our statistical methodology (TNNTg), this low percentage probably represents a lower-bound, with higher percentages, similar to those estimated previously reported (Ishikawa et al. 2017) being possible. Although fewer genes exhibited lower protein expression efficiency, those that did were significantly enriched in genes amplified by CNVs in each strain, suggesting that decreased protein expression efficiency may be a general mechanism of DC for amplified genes. This potential role in mediating DC is in agreement with observations of natural isolates of yeast with adaptive aneuploidy (Muenzner et al. 2024) and human cancers (Schukken and Sheltzer 2022). One possible mechanism of DC at the protein level is the targeted degradation of stoichiometrically imbalanced proteins involved in heteromeric protein complexes (Ishikawa et al. 2020). Intriguingly, dosage sensitive genes that incur a fitness cost at high copy-number levels were also identified as being enriched in proteins involved in protein complexes (Makanae et al. 2013), suggesting potential limits to the capacity for this mechanism to compensate for dosage imbalances.

We found genes with significantly different translation efficiencies to be significantly enriched in uORFs (Fig. 3b) consistent with the established role of these elements in the translational regulation of gene expression (Ingolia et al. 2009; Chew et al. 2016; Moro et al. 2021). We found that genes within CNVs were not specifically enriched in uORFs. However, all genes with significantly different translation efficiencies were significantly enriched for uORFs, regardless of being CNV amplified or not. This suggests that global changes in gene expression in response to adaptation may be translationally mediated by uORFs but that this may not be a mechanism of DC specific to CNV-amplified genes.

We also found that genes with significantly different translation efficiencies were also significantly enriched in binding motifs for the RBP Ssd1 (Fig. 3b) suggesting that it may also be involved in mediating changes in translation efficiency. Notably, *SSD1* has previously been shown to have a role in glucose starvation (Bresson et al. 2020), heat shock (Bayne et al. 2022), and quiescence (Miles et al. 2019). Ssd1 is capable of altering translation of bound RNA (Hogan et al. 2008; Ohyama et al. 2010) by preferentially binding to TLs and interfering with ribosome scanning (Bayne et al. 2022). We explored the potential for interaction between uORFs and Ssd1 and found that genes with uORFs are significantly enriched in Ssd1 binding motifs in their TLs and that these motifs have a closer proximity to the uORFs than expected by chance (supplementary fig. S12, Supplementary Material online). These findings suggest that Ssd1 may act as a stress responsive trans-acting factor driving uORF mediated translational regulation, potentially similar to the SXL protein binding that controls uORF translation of *msl2* in *D. melanogaster* (Medenbach et al. 2011).

These findings help contextualize the role of Ssd1 in CNV and aneuploid yeast strains. Previous studies have shown a much higher level of gene-specific DC in aneuploid yeast strains with a functional copy of *SSD1* (such as the S288c strain, as was used in this study) compared to yeast strains lacking a functioning *SSD1* (such as W303). This suggests that the importance of Ssd1 in mitigating the fitness defects of CNVs may be a consequence of the broader role Ssd1 plays in the translational regulation of gene expression under stress, and not specific to CNVs per se. Indeed, this may explain some of the observed but varying and incomplete overlap seen in Ssd1 targets, genes involved in the hypo-osmotic stress pathway (Bayne et al. 2022), CAGE (Tsai et al. 2019), and analyses that sought to identify the CNV and aneuploid stress response at the transcriptional level (Avecilla et al. 2023).

An unanticipated finding of our study is that the copy-number amplification of *GAP1* is also subject to dosage attenuation at multiple levels in several strains. We found *GAP1* has reduced transcription efficiency in all strains, significantly so in Trans and ODIRA_A strains. Similarly, it has significantly reduced translation efficiency in all strains. By contrast, *GAP1* has significantly higher protein efficiency in all strains except ComQuad. One potential biological explanation for this result is to consider that Gap1, a transmembrane protein (Merhi et al. 2011), is known to interact co-translationally with chaperones, including Shr3 (YDL212W) (Myronidi et al. 2023). Previous work suggests that in the absence of Shr3 many amino acid permeases including Gap1 will misfold and fail to exit the ER (Kota and Ljungdahl 2005), potentially forming aggregates leading to ER-associated degradation (Kota et al. 2007). We speculate that additional transcripts of *GAP1* enable lower densities of translating ribosomes, thereby decreasing the likelihood for ribosomal collisions and concomitant co-translational misfolding, resulting in a higher proportion of properly folded and functional proteins with fewer aggregates. In this case, although increased CN does not result in the expected increase in peptide synthesis, it yields increased production of proteins with the desired activity thereby conferring a positive fitness effect. Confirmation of this model would require further research on the cellular localization and transporter capacity of the Gap1 in ancestral and evolved strains.

Our study is not without its limitations. Notably, the number and type of strains evaluated limit the scope of our inferences and the generalizability of our results. Beyond that, technical limitations, such as ratio compression of TMT-labeled mass spectrometry data (Ting et al. 2011) and statistical tests of changes in expression across multiple levels make certain hypotheses hard to test. However, this work points to several avenues for ongoing research. While we have shown that gene expression, at the transcriptional and post-transcriptional levels is significantly different from the ancestor in adapted strains, it is unclear how rapidly these changes arise, how recurrent they may be, and how they change over the course of long-term evolution. Furthermore, the potential for RBPs to interact with uORFs and thereby alter downstream gene expression has only been investigated in few studies (Medenbach et al. 2011; Spealman et al. 2018). Our study points to the potential evolutionary significance of this mechanism motivating further investigation.

## Materials and Methods

### Strains and Growth Media

Strains evaluated in this paper were originally published in Lauer et al. (2018); they include both long-term experimentally evolved strains as well as their ancestor. The naming convention is as follows: Ancestor (DGY1657); Trans (DGY1726); ODIRA_A (DGY1735); ComQuad (DGY1741); and ODIRA_B (DGY1743). Trans and ODIRA_A are from population samples after ∼150 generations, ComQuad and ODIRA_B are from population samples after ∼250 generations. All strains are derived from FY4 (BY1747, supplementary fig. S1a, Supplementary Material online) and modified by the inclusion of a GFP and KanMX reporter cassette (supplementary fig. S1b, Supplementary Material online). All evolved strains were evolved in miniature chemostats under minimal glutamine growth media for either 150 or 250 generations, as described previously. For this study, strains were struck out on YEPD plates from −80 °C freezer stocks and grown for 2 d at 30 °C. Single colonies were selected based on the presence of fluorescence produced from the previously described mCitrine reporter (Lauer et al. 2018).

### Illumina Genomic DNA Sequencing

For short-read Illumina sequencing of strains we relied on previously generated data (Lauer et al. 2018; NCBI SRA accession SRP142330). Briefly, we performed genomic DNA extraction (Hoffman and Winston 1987), followed by quantification using SYBR Green I and standardized to 2.5 ng/μL. Libraries were constructed using Illumina Nextera tagmentation (Baym et al. 2015). Final concentrations were measured using SYBR Green I, fragment sizes were measured using Agilent TapeStation 2200 before being balanced and pooled. Illumina DNA libraries were sequenced on an Illumina NextSeq 500 using 2 × 75 paired-end protocol.

### Illumina Genomic DNA Alignment and Genotyping

All sequences were aligned against the Ensembl_R64.1.50 reference genome. We aligned reads using bwa mem (v0.7.17; Li and Durbin 2009) and generated BAM files using samtools (v1.14; Danecek et al. 2021). FASTQ files for all sequencing are available from the SRA (accession SRP142330).

We used GATK4 HaplotypeCaller (ver 4.1.9.0; Van der Auwera et al. 2013) to identify SNPs and indels in 4 evolved strains and their ancestor (DGY1657). HaplotypeCaller was run using a haploid ploidy with default settings against the Ensembl *S. cerevisiae* R64-1-1 reference genome. Variants were filtered using GATK VariantFiltration: SNPs (QD < 2.0, FS > 60.0, MQ < 30.0, SOR > 4.0, MQRankSum < −12.5, ReadPosRankSum < −8.0); Indels (QD < 2.0, FS > 200.0, SOR > 10.0); (supplementary table S12, Supplementary Material online). Variants were further filtered using Herança (v.0.8; https://github.com/pspealman/heranca), a lineage aware quality control script that filters variants more likely explained as sequencing errors (supplementary fig. S7, Supplementary Material online). Variants were annotated and predicted effects scored using Ensembl VEP (release 107; McLaren et al. 2016) default settings, *S. cerevisiae* R64-1-1, upstream/downstream distance (bp): 500. Potential structural and CNVs using Illumina paired-end data were identified using our custom analysis tool CVish (v1.1; Lauer et al. 2018; supplementary table S13, Supplementary Material online).

Potential SNV and indel identification of each strain was performed using GATK's HaplotypeCaller (v4.1.9.0; Van der Auwera et al. 2013) in single-sample mode and annotated using Ensembl VEP (release 107; McLaren et al. 2016). These are reported in the supplement (supplementary table S12, Supplementary Material online). Potential structural and CNVs using Illumina paired-end data were identified using our custom analysis tool CVish (v1.1; Lauer et al. 2018; supplementary table S13, Supplementary Material online).

### Nanopore Genomic DNA Sequencing, Alignment, and Genotyping

All yeast strains were grown to >1 × 10^7^ cells/mL in 300 mL glutamine minimal media. Genomic DNA from each strain was extracted using Qiagen 20/G Genomic tips from ∼1.5 × 10^9^ cells using the manufacturer's protocol.

All genomic DNA was barcoded using Oxford-Nanopore's native barcoding genomic DNA kit (EXP-NBD104), adapters were added using the ligation sequencing kit (SQK-LSK109). The manufacturer's protocol (versions NBE_9065_v109_revB_23May2018 and NBE_9065_V109_revP_14Aug2019) was followed with the following exceptions: incubation times for enzymatic repair step were increased to 15 min. All Agencourt AMPure XP beads were incubated for 30 min at 37 °C before elution. Adapter ligation time was increased to 10 min. Multiplexed libraries were loaded on MinION flowcells (FLO-MIN106D R9) and run on a MinION sequencer (MIN-101B) (Quinlan and Hall 2010).

All sequences were aligned against the Ensembl_R64.1.50 reference genome (Cunningham et al. 2022). Nanopore long-read sequences were aligned using minimap2 (v2.22; Li 2018), with variant detection using sniffles2 (v2.0.6; Li 2018; Sedlazeck et al. 2018), potential ODIRA CNV breakpoints were evaluated with mugio (v1.7; Spealman et al. 2020). DNA depth across genomes was calculated per nucleotide using bedtools “genomecov” (ver 2.29.2; Quinlan and Hall 2010). These were used in conjunction with the CNV breakpoints identified by CVish to manually reconcile the CNV topologies for each strain (supplementary fig. S1 and table S1, Supplementary Material online).

### DNA Reads and Depth Estimation

We identified ∼15.4% of genes in the ancestral strain had long-read DNA read-depth not near 1 copy. Some of these are known deletions (*MATALPHA* and *SRD1*) or are regions with known properties of known CN expansion (*CUP1* region, ENA region, and ASP region) or transposons. To prevent the over or underestimation of expected expression in evolved strains, we manually analyzed the locus and flanking regions for signs of CNV breakpoints using both short and long-reads. If no evidence was found for a breakpoint, the locus was assigned a copy number of 1 (supplementary table S1, Supplementary Material online).

### Growth Conditions and Cell Harvesting

Colonies were grown overnight on YPD then used to inoculate 500 mL of minimal glutamine growth media in chemostats. These were then grown to saturation (∼48 h ∼1e7 cells/mL, measured using Coulter Counter) at 30 °C in aerobic conditions. After saturation was achieved chemostats were switched to continuous mode with an inflow rate of fresh minimal glutamine growth media at a rate of 0.12 h/L (corresponding to a population doubling time of ∼5.8 h). This was maintained for 24 h before the cells were harvested. This was performed in duplicate for each strain for the simultaneous extraction of RNA and RPFs. This same procedure was also performed separately for the 5 replicates used for the generation of material for mass spectrometry.

### RNA-seq and Ribosome Profiling

Cells were harvested following previously described methods (Spealman et al. 2016). Briefly, cycloheximide was added to the media to a final concentration of 100 μg/L and incubated for 2 min, before being separated from the media using rapid vacuum filtration using Millipore 0.22 μm filters. Cells were resuspended from filters using polysome lysis buffer with cycloheximide (CHX) and then flash frozen immediately in liquid nitrogen before being transferred and stored at −80 °C. RNA-seq and ribosome profiling was performed by TB-SEQ, Inc. (Palo Alto, CA). As per (McGlincy and Ingolia 2017), cells were lysed in polysome lysis buffer with CHX, cells for each sample were split into RNA and RPF aliquots. For RPF aliquots, RNase I was used to digest polysomes, monosomes were purified using a sucrose cushion. Fragment size selection of 25 to 32 nucleotides was performed using a polyacrylamide gel. No ribosomal depletion or poly-A tail selection was made to either RPF or RNA libraries. Fragment size and concentrations were measured using bioanalyzer and qubit before sequencing using Illumina NovaSeq6000, single read, 1 × 50 cycles.

### Mass Spectrometry and Analysis

For the generation of material for MS 5 biological replicates for each strain were grown in chemostats as described above. Cells were collected from the chemostats using the same techniques described above. TMT-labeling and LC-MS were performed by Proteomics Laboratory, NYU Langone Medical Center (New York, NY). We used 16Plex TMT labeling to enable pooling of peptides from 16 samples (Ch1-16) for offline fractionation and LC-MS/MS detection and quantification (supplementary figures, Supplementary Material online). Five replicates were generated for each strain and the total run was performed in 2 batches (“A” and “B”). Ch1 was used as the common reference standard, an equimolar mix of all the samples, labeled with TMT Ch1. All samples were lysed using a TFA approach (Doellinger et al. 2020). Both batches were fractionated by offline HPLC chromatography using reverse phase C18 stationary phase at high pH. Peptides from collected fractions were separated by online HPLC on C18 at low pH coupled to the MS instrument.

### Gene Expression Analysis

RNA and RPFs were aligned using STAR (ver 2.7.6a; Dobin et al. 2013) filtering for known nonpolyadenylated ncRNA (snRNA, rRNA, and tRNA) using Ensembl_R64.1.1 ncRNA fasta (supplementary File SF1, Supplementary Material online). Counts per gene were calculated using bedtools (v2.29.2; Quinlan and Hall 2010) coverage -counts -s -b against Saccharomyces_cerevisiae.R64-1-1.50.gff3. For mass spectrometry, the spectra were analyzed using MaxQuant (v2.1.0.0; Tyanova et al. 2016a) with parameters described previously (Yu et al. 2020). Parameter and configuration files are provided as supplemental file (supplementary File SF2, SF3, Supplementary Material online).

Spearman rho correlations between replicates for each level of expression for each strain were calculated (supplementary table S2, Supplementary Material online), for RNA median rho between replicates (*rho* = 0.9878), RPF (*rho* = 0.9879), and MS (*rho* = 0.9965), which is sufficiently high for replicates. Between levels (but within the same strain) we found the median rho for RNA to RPF (*rho* = 0.6271), RPF to MS (*rho* = 0.6012), and RNA to MS (*rho* = 0.5453). We also compared between strains (supplementary table S3, Supplementary Material online) and found the strains to be very well correlated with the median rho for RNA (*rho* = 0.9396), RPF (*rho* = 0.9258), and MS (*rho* = 0.9915).

Differential gene expression analysis for RNA and RPF abundances were calculated DESeq2 (v1.6.3; Love et al. 2014). Results of DESeq2 are available as supplemental files (**RNA,**  supplementary table S4, Supplementary Material online**; RPF,**  supplementary table S5, Supplementary Material online). Significance is defined as BH adjusted *P*-value < 0.05.

Differential gene expression analysis for mass spectrometry data was performed using Perseus (v1.6.15.0; Tyanova et al. 2016b). Data preprocessing and normalization procedures were performed as described (Yu et al. 2020). The statistical test for significance in differential abundance is Welch's *t*-test of the difference between the normalized protein group abundances in each strain relative to the ancestor. Significance here is defined as *q*-value < 0.05 using BH multiple hypothesis test correction (i.e. BH adj. *P*-value < 0.05; supplementary table S6, Supplementary Material online). Volcano plots indicating the protein abundances of CNV in each strain are in the supplemental file (supplementary figs. S13 to S16, Supplementary Material online).

Heatmap and clustering analysis for the purposes of data visualization was performed using (*pheatmap*; R Core Team 2019) and (*cutree*; R Core Team 2019), respectively. A full version of the heatmap (supplementary fig. S9, Supplementary Material online) and cluster assignment (supplementary table S7, Supplementary Material online) are available as Supplementary material. GO term enrichment was calculated using Yeastmine (Balakrishnan et al. 2012) with significance cutoff at BH adj-*P*-value < 0.05. Redundant terms were then reduced using REVIGO (Supek et al. 2011) using the “Tiny” option, UNIPROT *Saccharomyces cerevisiae* S288c database, and SimRel semantic similarity measure. Heterodimer protein complexes are from a manually curated list from (Taggart and Li 2018).

All Venn diagrams were originally visualized using Venny (v2.1.0, https://bioinfogp.cnb.csic.es/tools/venny/), proportional Venn diagrams were generated using DeepVenn (Hulsen 2022). All RNA, RPF expression data were visualized using IGV browser (v2.9.4; Robinson et al. 2011).

For quality control purposes, we set an arbitrary low expression threshold. Genes were required to not be transposon element associated genes, have at least 100 aligned RNA reads, 10 RPFs, and have a significant majority peptide fragment detected by MS. This reduced our gene set to 4,289 genes in total. Genes were further filtered, on a strain by strain basis, if the CNV breakpoint was found to disrupt their expression (supplementary figs. S2 to S6, Supplementary Material online).

Importantly, technical limitations involving chemostat size, cell concentration, and the material requirements necessary for library generation, cells collected for use in the generation of the RNA and RPF data were grown in separate chemostats than the cells used for the generation of the MS materials. Although this may have introduced batch effects several lines of evidence suggest otherwise, such as the high degree of overlap in significantly differentially expressed genes at each level (supplementary fig. S9, Supplementary Material online), the global similarity in expression across all levels (supplementary fig. S10, Supplementary Material online), and that the observed correlation of expression between MS and RNA is (median *rho* = 0.55), it is similar to previous observations (de Godoy et al. 2008; Vogel and Marcotte 2012).

### Transcription Efficiencies Calculated as Observed Versus Expected

Transcription efficiency was calculated using evolved to ancestor strain pairwise tests of observed to expected RNA abundance using DESeq2, using 2 replicates each. Expected RNA abundances were calculated using a custom script that multiplies the observed abundance in ancestor by the copy number of that gene in the evolved strain. Differential abundance analysis was performed on both unnormalized observed and unnormalized expected RNA-seq reads using DESeq2 (v1.6.3; Love et al. 2014). Results of DESeq2 are available as a supplemental file (supplementary table S8, Supplementary Material online). Significance here is defined as BH adj. *P*-value < 0.05 as calculated by DESeq2.

### Translation Efficiencies Calculated Using Ratiometrics and FET

Translation efficiency was calculated using evolved strain to ancestor strain pairwise tests of RPF/RNA ratios. RPF and RNA abundances are first normalized using TPM (supplementary fig. S17b and e, Supplementary Material online). Replicates were combined by taking the median RPF and median RNA separately before assessment. Evolved RPF/RNA ratios per gene were compared to the ancestral RPF/RNA ratios using FET. Results of FET evaluation of translation efficiency are available as a supplemental file (supplementary table S9, Supplementary Material online). Note, genes with significantly different translation efficiencies described in the results section were identified using FET not TNNTg.

### Protein Efficiencies and Scale Independent Tests

Ideally, protein expression efficiency would be calculated using a ratiometric method similar to translation efficiency. However, while ratiometric analysis is robust for similarly scaled data, it may generate spurious results for data with excessively different scales. So while ratiometric analysis of RPF and RNA ratios is sensible, scaling becomes a greater concern when comparing RPFs to MS intensities which can be 2 to 4 magnitudes apart. One solution is to use outlier detection methods that are robust to differences in scale between input variables, such as GLMs.

Because MS intensities are continuous values and not counting abundances we selected a Gaussian model (*glm*, family = “gaussian”; R Core Team 2019) fit to the untransformed expression values in both the evolved and ancestor. For translation efficiency: Evolved RPF ∼ (Evolved RNA * Ancestor RNA * Ancestor RPF). For protein efficiency: Evolved MS ∼ (Evolved RPF * Ancestor MS * Ancestor RPF). Outliers are then identified as those with standardized residuals (*rstandard*; R Core Team 2019) >1.96 (Kuhnt and Pawlitschko 2005). To better understand the performance of the GLM, we compared GLM performance to FET using translation efficiency data (e.g. RPF, RNA). Because of the large number of true negatives (∼4,000 genes) we chose to measure performance using the F1 score, or harmonic mean of precision and recall, which is true negative agnostic (Van Rijsbergen 1977). F1 scores range from [0 to 1] with lower scores representing few true positives relative to false positives and false negatives, while a high score represents a higher rate of true positives. We found the GLM performed well (F1 score = 0.32) with a very low false-positive detection rate (FPR = 0.008). This, combined with the high false negative rate (FNR = 0.78) suggests an accurate but overly conservative test (supplementary fig. S18, Supplementary Material online).

### Near-Neighbor Differential Test on Transformed Data

In addition to GLMs which are robust to differences in variable scale, another solution is to first transform the measurements to similar scales before proceeding with a differential test.

While there are many approaches to data transformation, we opted to use Box-Cox power transform (supplementary fig. S17c, f and i, Supplementary Material online) as implemented in scikit-learn as it retains meaningful features of the original distribution and results in a more Gaussian final distribution (Pedregosa et al. 2011). We next transformed the data by MinMax scaling to a range of (0,1] (Pedregosa et al. 2011) so that all values greater than zero. While the transformed data are now at a similar scale it is no longer count data and may break many of the assumptions FET requires. Instead, we evaluate the transformed data using a TNNT to identify local outliers.

For the purposes of evaluation, we can compare performance of this method to the results of FET performed on translation efficiency. In this case “efficiency” is the ratio of RPF to RNA (in power transformed units). However, when this method is applied to protein expression efficiency it refers to the ratio of MS to RPF (in power transformed units).

First, we rank all genes using the distance between the evolved and ancestral efficiency ratios (supplementary fig. S19a, Supplementary Material online). Then, we score each gene by taking the ratio of the evolved efficiency to the ancestral efficiency. Ranking first by distance helps to minimize spurious detections resulting from comparing ratios with small numbers (e.g. 1000/100 is not equivalent to 10/1). To determine if this score is greater than expected by chance, we next generate a neighborhood of the nearest 10% of genes of similar rank. This near-neighborhood is then randomly subsampled to count the number of times the given score is met or exceeded by similarly ranked genes. This permutation of the near-neighbor is conducted 5 times for each gene. This observed over expected frequency is then used to estimate an FDR wherein an FDR of 0.05 means that 5% of the genes out of a random sampling of similarly ranked genes met or exceeded the score being evaluated. Furthermore, we require that the variance in expression between replicates cannot exceed the distance between strains.

One way to evaluate TNNT is to benchmark it against FET. In a performance comparison to FET to TPM normalized translation efficiency ratios, we find TNNT has a FPR of 0.01, a FNR of 0.40 and a F1 score of 0.63 (supplementary fig. S19f, Supplementary Material online). The combination of high F1 score, high FNR, and low FPR suggests that TNNT is less conservative than the GLM test used above but is still sacrificing sensitivity for precision when compared to FET. This performance is also robust to analyses involving fewer replicates as evaluated by reducing the number of MS replicates from 5 to 2, balancing these with the 2 replicates of RPF data (supplementary fig. S21, Supplementary Material online).

Additively combining the results of the GLM test and TNNT (now referred to as TNNTg) adds additional power to the TNNT method (supplementary fig. S20, Supplementary Material online) without sacrificing precision (F1 = 0.63, FPR = 0.018, FNR = 0.43). This combined method is used for protein expression efficiency analysis.

### Protein Expression Efficiency Calculated Using TNNTg

For each evolved strain, we compared the protein expression efficiency ratio (transformed MS intensity/transformed RPF abundance) to the Ancestor ratio (supplementary fig. S17f and i, Supplementary Material online). Replicates were combined by taking the median MS and median RPF separately before assessment as a ratio. Significant differences in protein expression efficiency between the evolved and ancestor strains were calculated using the TNNTg method (supplementary fig. S20, Supplementary Material online); such that a gene is either an outlier relative to near-neighbors using transformed data at FDR < 0.05 or is an outlier with a standardized residual >1.96, as calculated by GLM. Results of the TNNTg evaluation of translation efficiency are available as a supplemental file (supplementary table S11, Supplementary Material online).

### Identification of High Confidence Predicted uORFs

Because uORF activity is known to be condition sensitive (Hinnebusch 2005; Moro et al. 2021) and no previous study has been conducted using glutamine-limited growth media or CNVs, we sought to identify uORFs using our ribosome profiling data. To identify ribosome occupied uORFs in these datasets, we developed uORFish (ver.1.3, github.com/pspealman/uorfish), a deep neural network uORF predictor trained on “validated” uORFs from Spealman et al. (2018), Eisenberg et al. (2020), and May et al. (2023).

To prevent training on uORFs that are not active under the conditions used, validated uORFs are first curated. A random null model for each category of uORF is generated by shuffling nucleotide triplets within TLs and scoring the resultant uORFs (supplementary fig. S10, Supplementary Material online). Curated uORFs must score above the top 5% of random null generated uORFs. These curated, validated, uORFs are then used to train a DNN (pytorch; Paszke et al. 2019) per ribosome profiling replicate (supplementary fig. S11, Supplementary Material online). High confidence predictions are defined as uORFs predicted independently for each replicate. Comparison of results using triplicate data (Nedialkova and Leidel 2015) suggest sufficient performance with 2 replicates for the identification of condition specific uORF utilization (supplementary fig. S11f, Supplementary Material online). Predicted scores for all candidate uORFs with scores >0.5 are available as supplemental files (supplementary table S14, Supplementary Material online).

### SSD1 Motif Analysis

To identify genes enriched in SSD1 motifs in their TLs, we first generated a list of TLs for all genes using the Yeastmine (Balakrishnan et al. 2012) annotated “Five Prime UTR” (Pelechano et al. 2013) or from (Spealman et al. 2018), taking the longest recorded instance. Combined these encompassed 6,119 genes, 6,080 of which exceeded the length of the motif. We converted these to fasta using bedtools “getfasta” (ver2.29.2; Quinlan and Hall 2010). These were then scanned for perfect matches to the CNYUCNYU motif (Bayne et al. 2022), irrespective of strand. The genome-wide background rate was calculated per nucleotide as 2.39e−3, the background rate using only TLs was 2.91e−3 per nucleotide. Significance was calculated using FET, using the proportional ratio of (observed hits of TL/length of TL) versus (total hits/total length), (supplementary table S15, Supplementary Material online). We found that of the 219 genes that were significantly enriched in SSD1 motifs, 163 were present in our data set.

## Supplementary Material

msaf005_Supplementary_Data

## Data Availability

All data are publicly available. Illumina short-reads are available on NCBI SRA (PRJNA451489). ONT Nanopore long-reads are available on NCBI SRA (PRJNA591579). RNA-seq (GSE246093) and ribosome profiling (GSE246094) data are available through NCBI GEO. Mass spectrometry data is available through PRIDE (PXD046587). All scripts and data used in the analysis included in this paper are available via GitHub: https://github.com/GreshamLab/adaptive_gene_expression.
