# Supplementary material for: Multilevel Gene Expression Changes in Lineages Containing Adaptive Copy Number Variants
Source: Mol Biol Evol. 2025 Jan 23;42(2):msaf005. doi: 10.1093/molbev/msaf005 (PMC11789944; doi:10.1093/molbev/msaf005)
Supplement: msaf005_Supplementary_Data [file msaf005_supplementary_data.zip › Supplemental_Materials.pdf]

Supplemental figures:

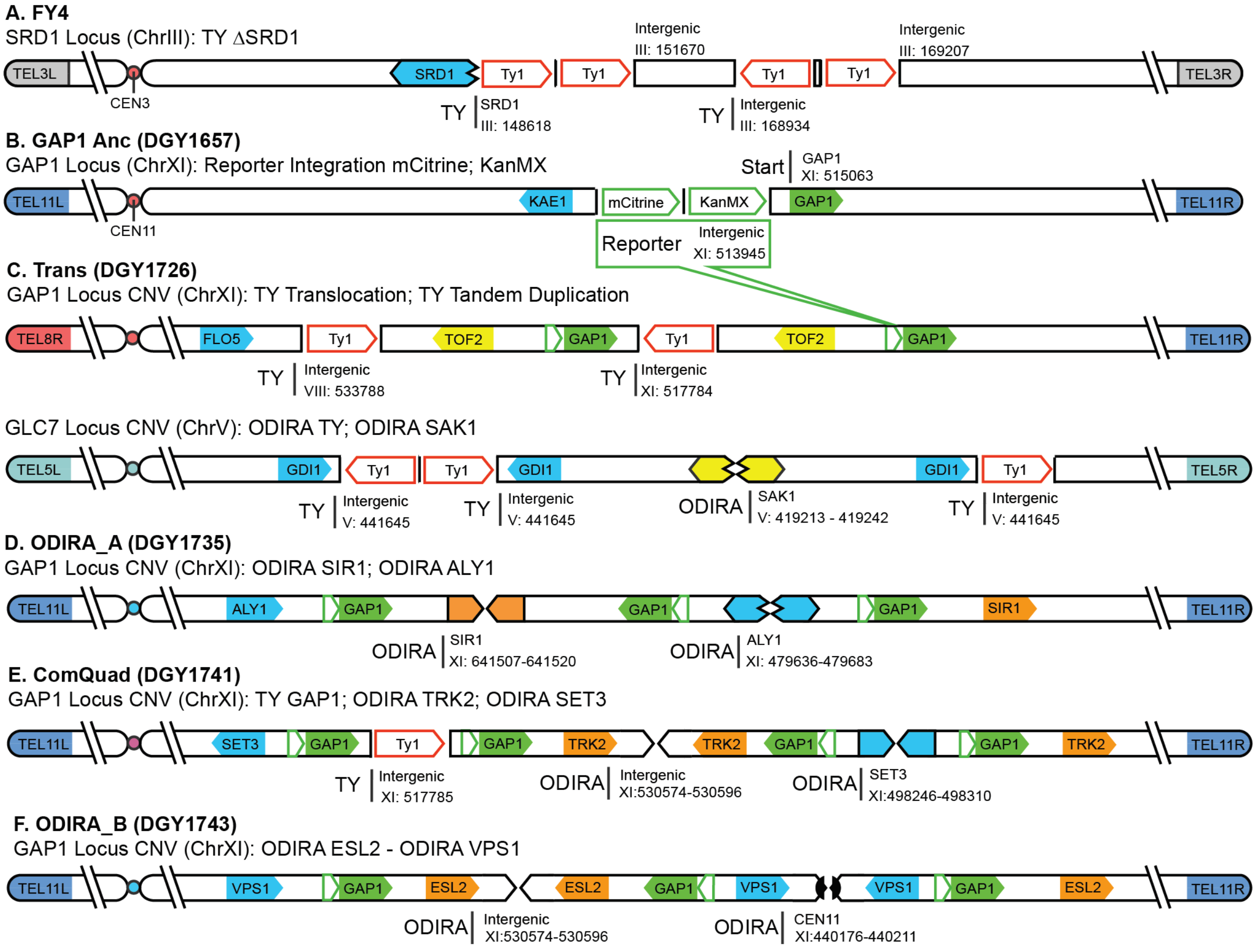

Figure S1. Schematic of CNV Topology

(**A**). Diagram showing the disruption of *SRD1* in FY4 (**B**). Diagram showing the integration of the reporter in DGY1657 (**C**), the reporter is represented by a green box in all subsequent diagrams. Topology diagrams for evolved strains indicating CNV breakpoints, orientations, and the occurrence of transposon events **C-F**). CNV breakpoints are annotated with their most likely mechanism: transposon-yeast (red arrow), origin-dependent inverted-repeat amplification (ODIRA). Gene copy-number values for each strain are available in Supplementary Table 1.

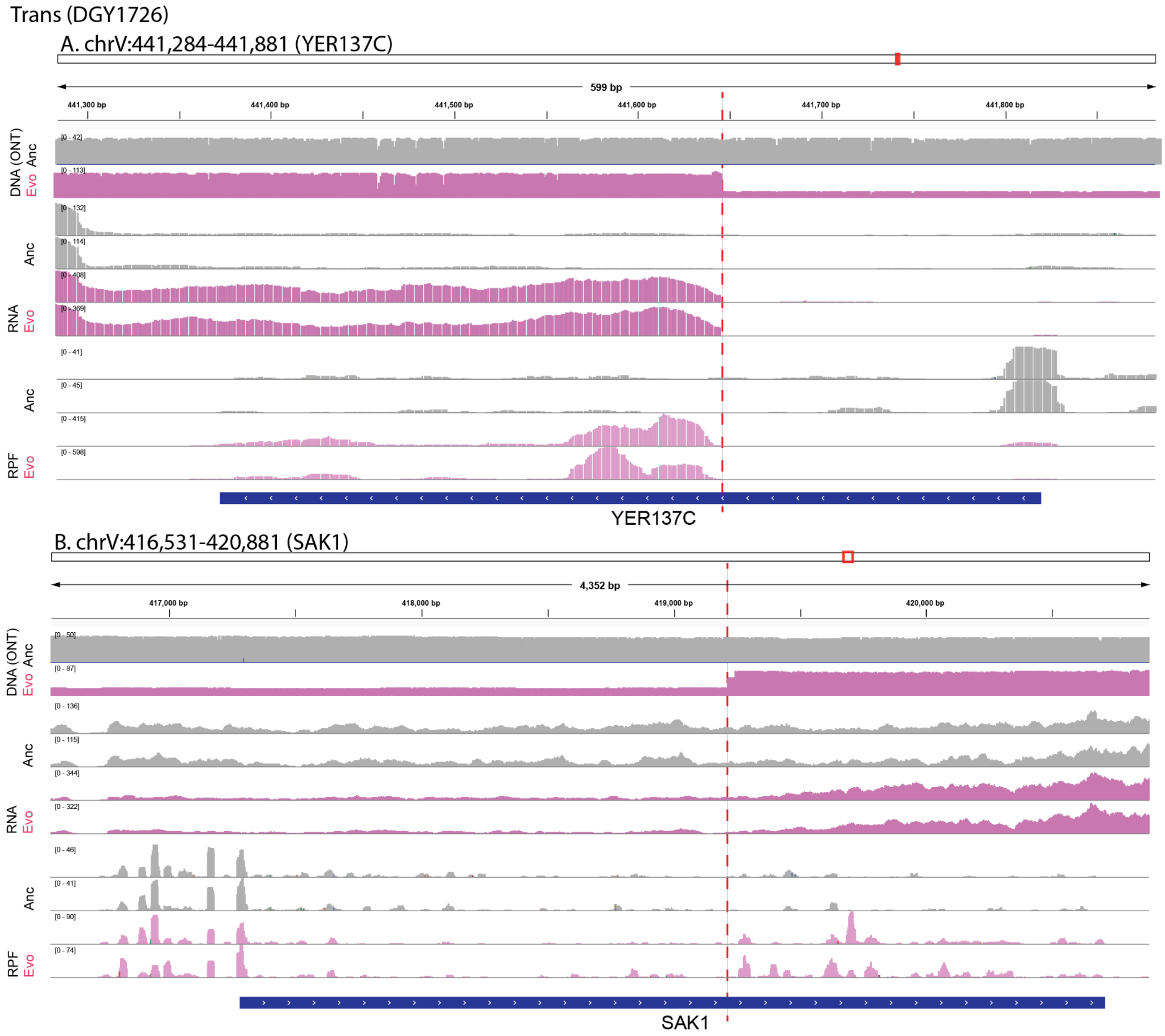

Figure S2. CNV breakpoint in association with RNA and RPF data for Trans (DGY1726) on chromosome V. IGV (Robinson et al. 2011) generated tracks showing Ancestor (Grey, DGY1657) and Evolved (Purple, DGY1726) tracks. Long-read DNA (ONT) is the top two tracks followed by RNAseq data (in replicate), and ribosome protected footprints (RPF, in replicate). Vertical dashed line shows the proposed CNV breakpoint. (**A**) Likely breakpoint inside *YER137C* with disruption to RNA and RPF expression. This gene is removed from the analysis. (**B**). Likely breakpoint inside of *SAK1* with disruption to RNA and RPF expression. This gene is removed from the analysis.

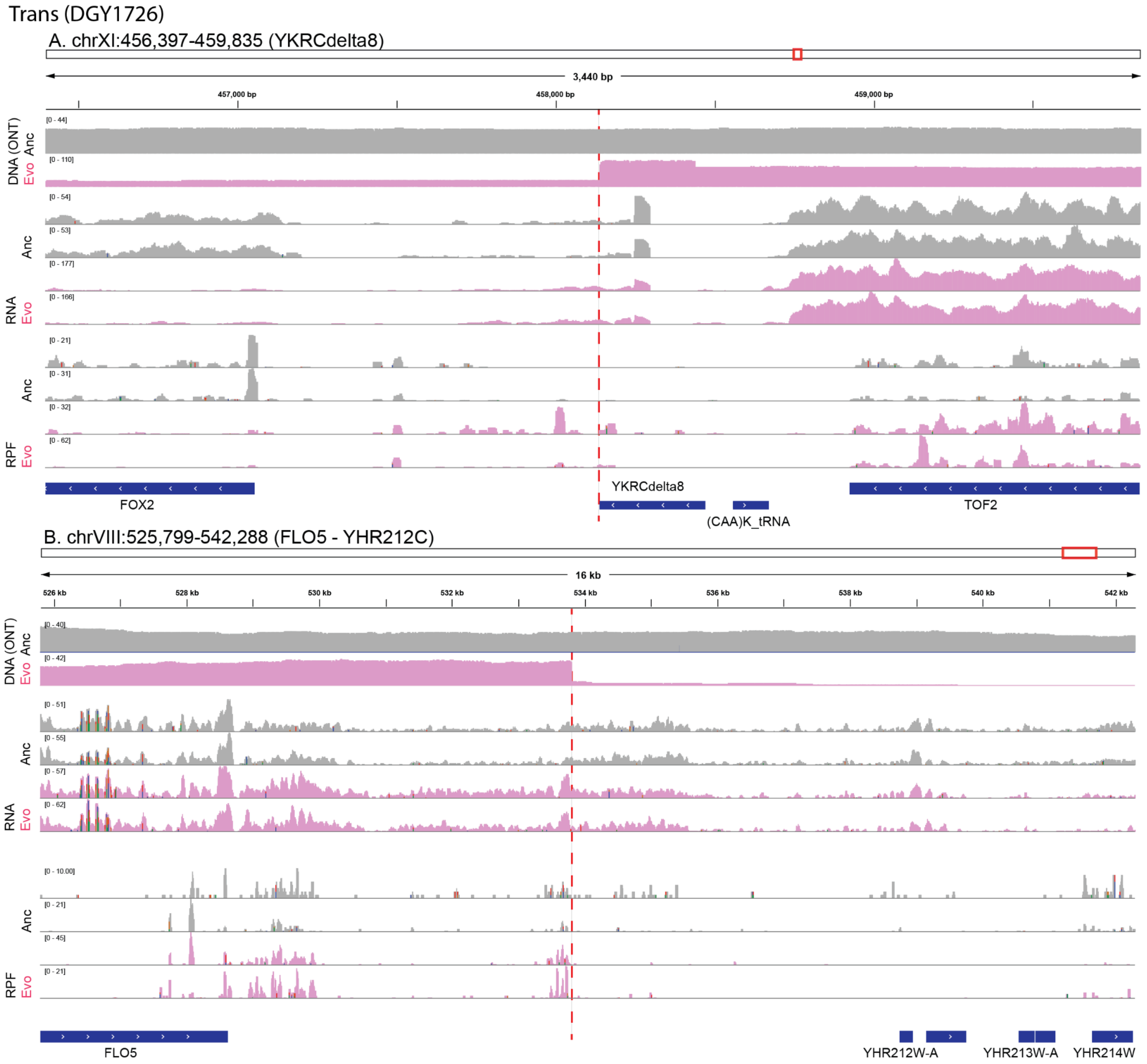

Figure S3. CNV breakpoint in association with RNA and RPF data for Trans (DGY1726) on chromosome XI and chromosome VIII. IGV (Robinson et al. 2011) generated tracks showing Ancestor (Grey, DGY1657) and Evolved (Pink, DGY1726) tracks. Long-read DNA (Oxford Nanopore Technology ONT) is the top two tracks followed by RNAseq data (in replicate), and ribosome protected footprints (RPF, in replicate). Vertical dashed line shows the proposed CNV breakpoint. (**A**) CNV with TY1 mediated translocation, showing YKRCdelta8 as the likely breakpoint. No obvious disruption to expression near locus. (**B**). TY1 mediated translocation reception site, VIII:533788, intergenic between *FLO5* and *YHR212C*. No obvious disruption to expression near locus.

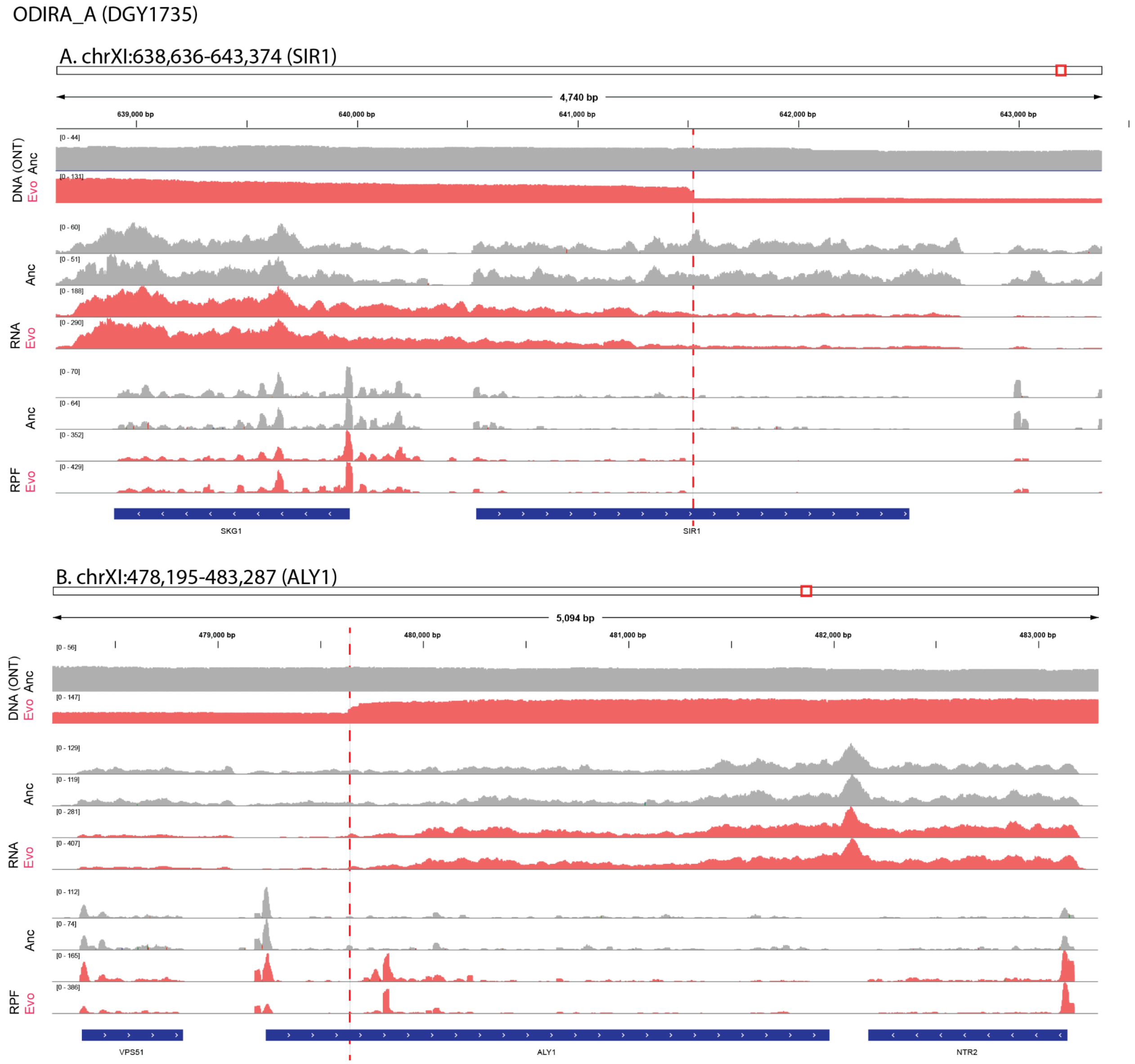

Figure S4. CNV breakpoint in association with RNA and RPF data for ODIRA_A (DGY1735) on chromosome XI. IGV (Robinson et al. 2011) generated tracks showing Ancestor (Grey, DGY1657) and Evolved (Red, DGY1735) tracks. Long-read DNA (ONT) is the top two tracks followed by RNAseq data (in replicate), and ribosome protected footprints (RPF, in replicate). Vertical dashed line shows the proposed CNV breakpoint. (**A**) Likely breakpoint inside *SIR1* with disruption to RNA and RPF expression. This gene is removed from the analysis. Likely disruption of RNA and upstream RPF of flanking *SKG1* as well. (**B**). Likely breakpoint inside of *ALY1* with disruption to RNA and RPF expression. This gene is removed from the analysis.

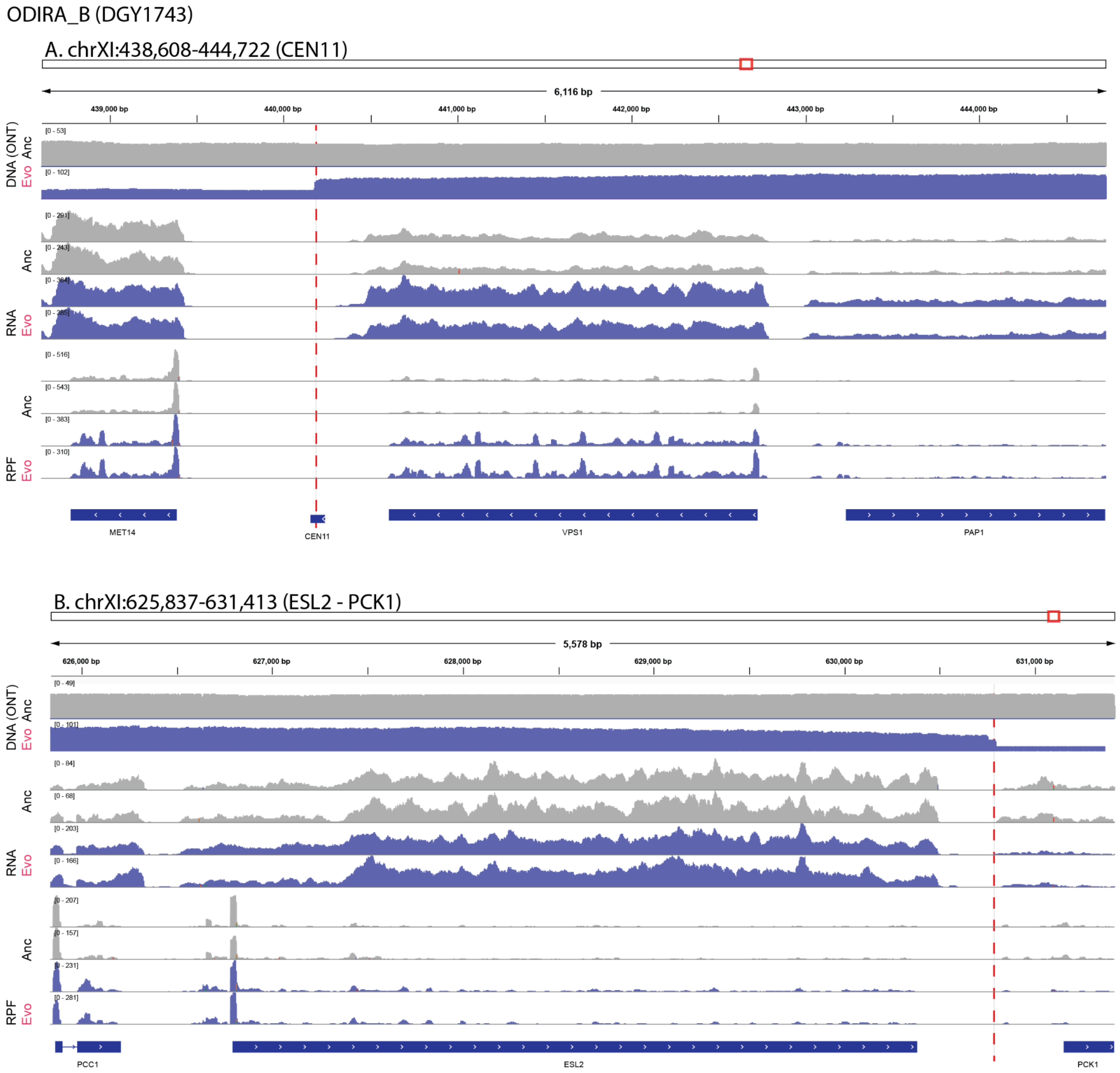

Figure S5. CNV breakpoint in association with RNA and RPF data for ODIRA_B (DGY1743) on chromosome XI. IGV (Robinson et al. 2011) generated tracks showing Ancestor (Grey, DGY1657) and Evolved (Blue, DGY1743) tracks. Long-read DNA (ONT) is the top two tracks followed by RNAseq data (in replicate), and ribosome protected footprints (RPF, in replicate). Vertical dashed line shows the proposed CNV breakpoint.(**A**). Likely breakpoint within CEN11. No obvious disruption to expression near locus. (**B**). Likely breakpoint within the intergenic region between *ESL2* and *PCK1*. No obvious disruption to expression near locus.

ComQuad (DGY1741)

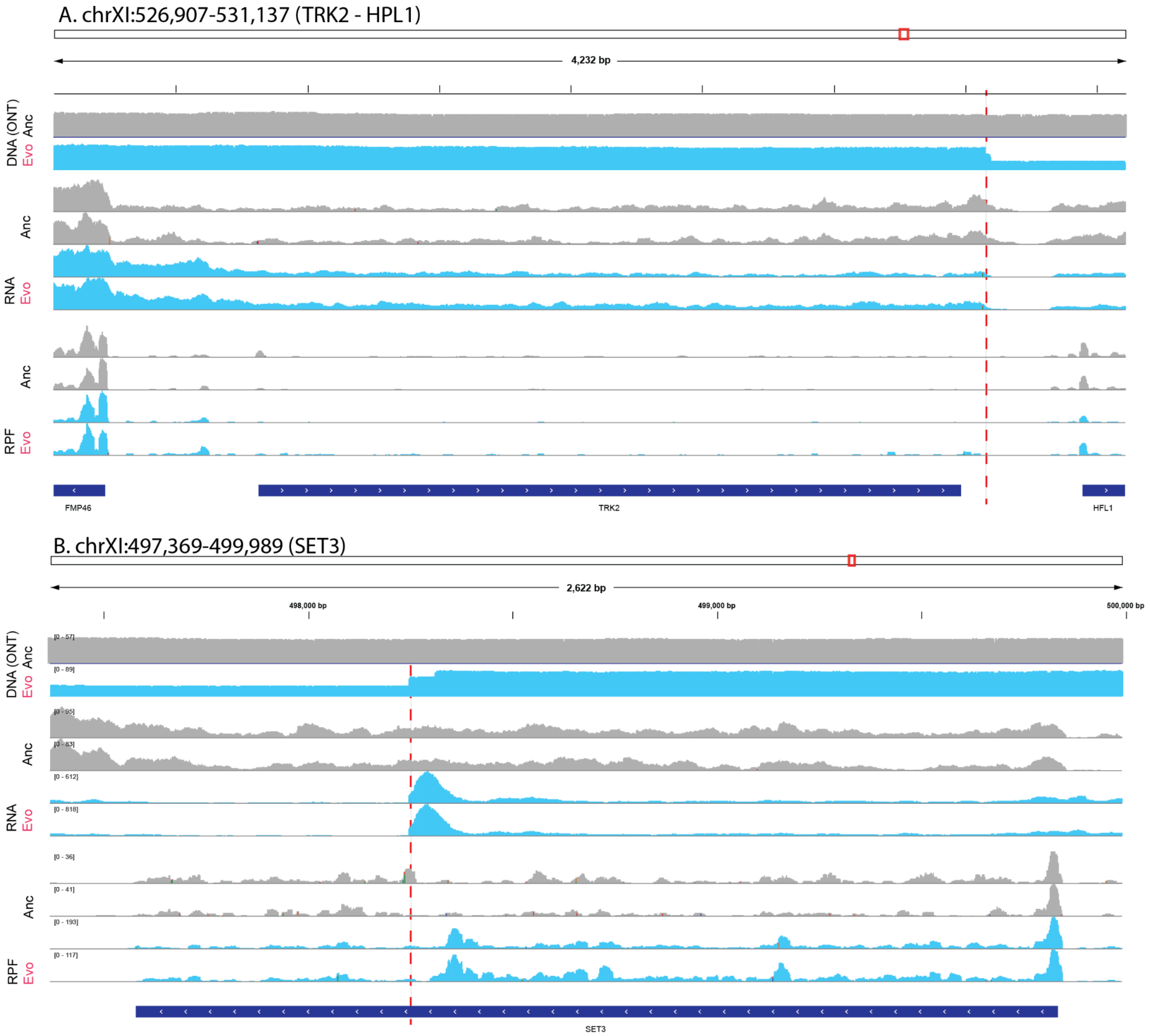

Figure S6. CNV breakpoint in association with RNA and RPF data for ComQuad (DGY1741) on chromosome XI. IGV (Robinson et al. 2011) generated tracks showing Ancestor (Grey, DGY1657) and Evolved (Blue, DGY1741) tracks. Long-read DNA (ONT) is the top two tracks followed by RNAseq data (in replicate), and ribosome protected footprints (RPF, in replicate). Vertical dashed line shows the proposed CNV breakpoint. (**A**) Likely breakpoint immediately downstream of *TRK2*. No obvious disruption to expression near locus, although the 3'UTR may be truncated. (**B**). Likely breakpoint inside of *SET3* with disruption to RNA and RPF expression. This gene is removed from the analysis.

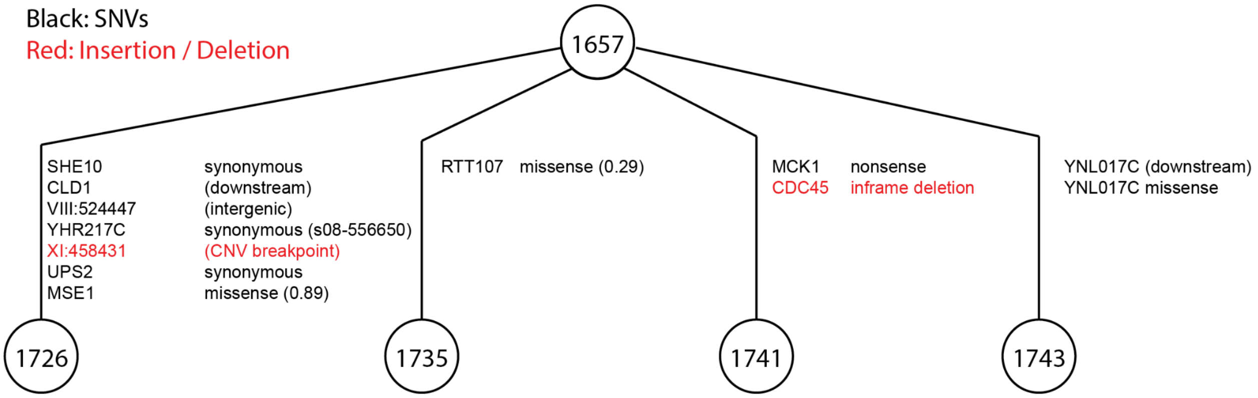

Figure S7: Mutations in evolved strains

Schematic representing the variants in the evolved strains versus their ancestor. Each variant in black is a SNP while variants in red are Indels. Variants within the CDS of a gene are marked as synonymous, nonsense, or missense. Missense variants also have an estimated mutational effect value (SIFT) when possible with lower values being more severe. In only one case was a CNV breakpoint identified using GATK (XI:458431, DGY1726). All variants are novel (ie. not in Ensembl Variant Catalog) except for the synonymous mutation of YHR217C in DGY1726

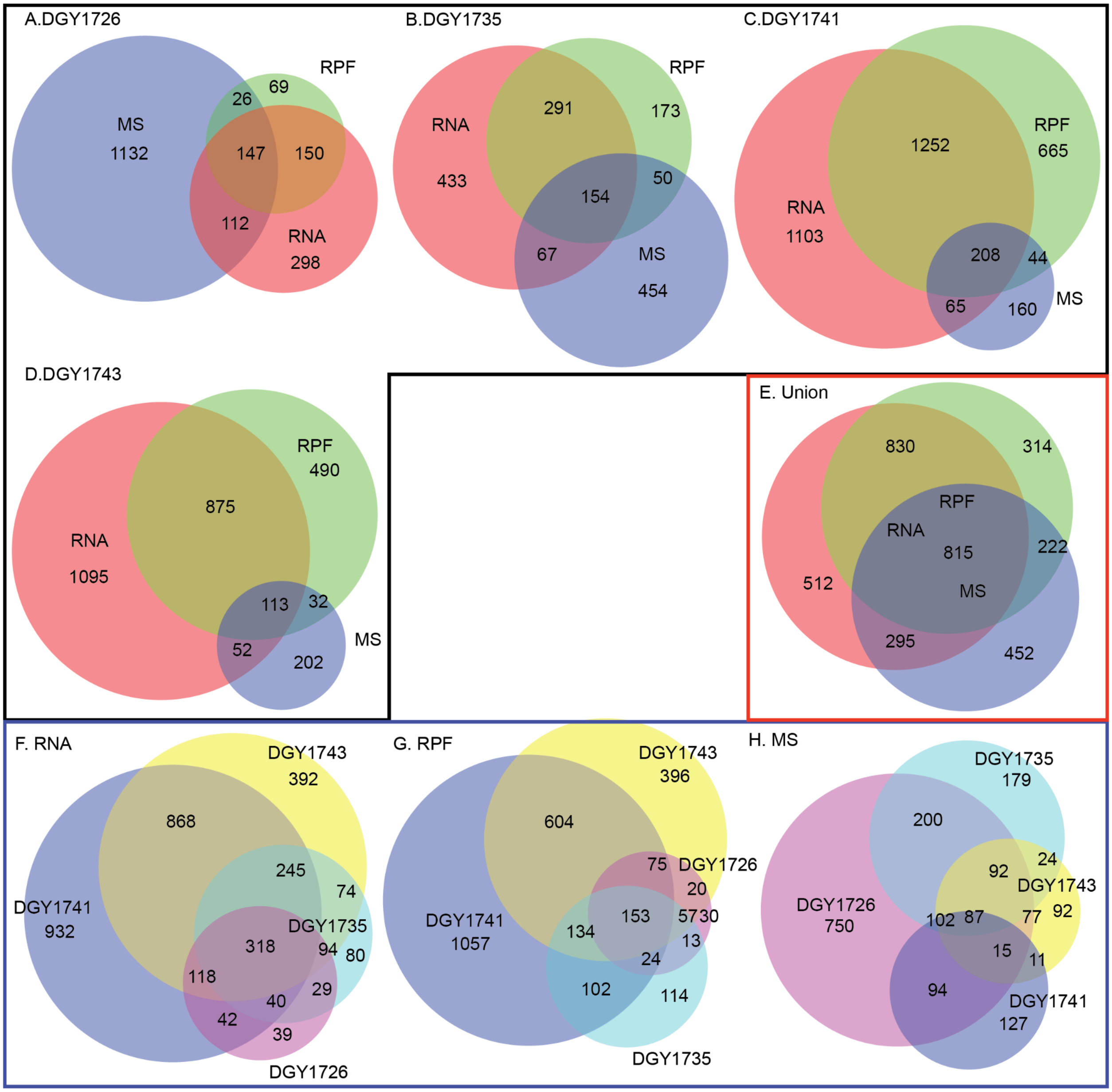

Figure S8: Differential gene abundances for each strain and level of expression

Venn diagrams depicting the abundances of significantly differentially expressed genes for each strain (**black box A, B, C, D**). Differential abundances for RNA and RPF used DESeq2 (Benjamin-Hochberg (BH) correction adj.p-value < 0.05); MS used Welch's t-test (BH, adj.p-value < 0.05). The union of these genes at each level (**red box, E**), after removing genes below quality control threshold (methods), as reported in the main text. Venn diagrams depict the overlap of significantly differentially expressed genes between strains at each level of expression (**blue box, F, G, H**).

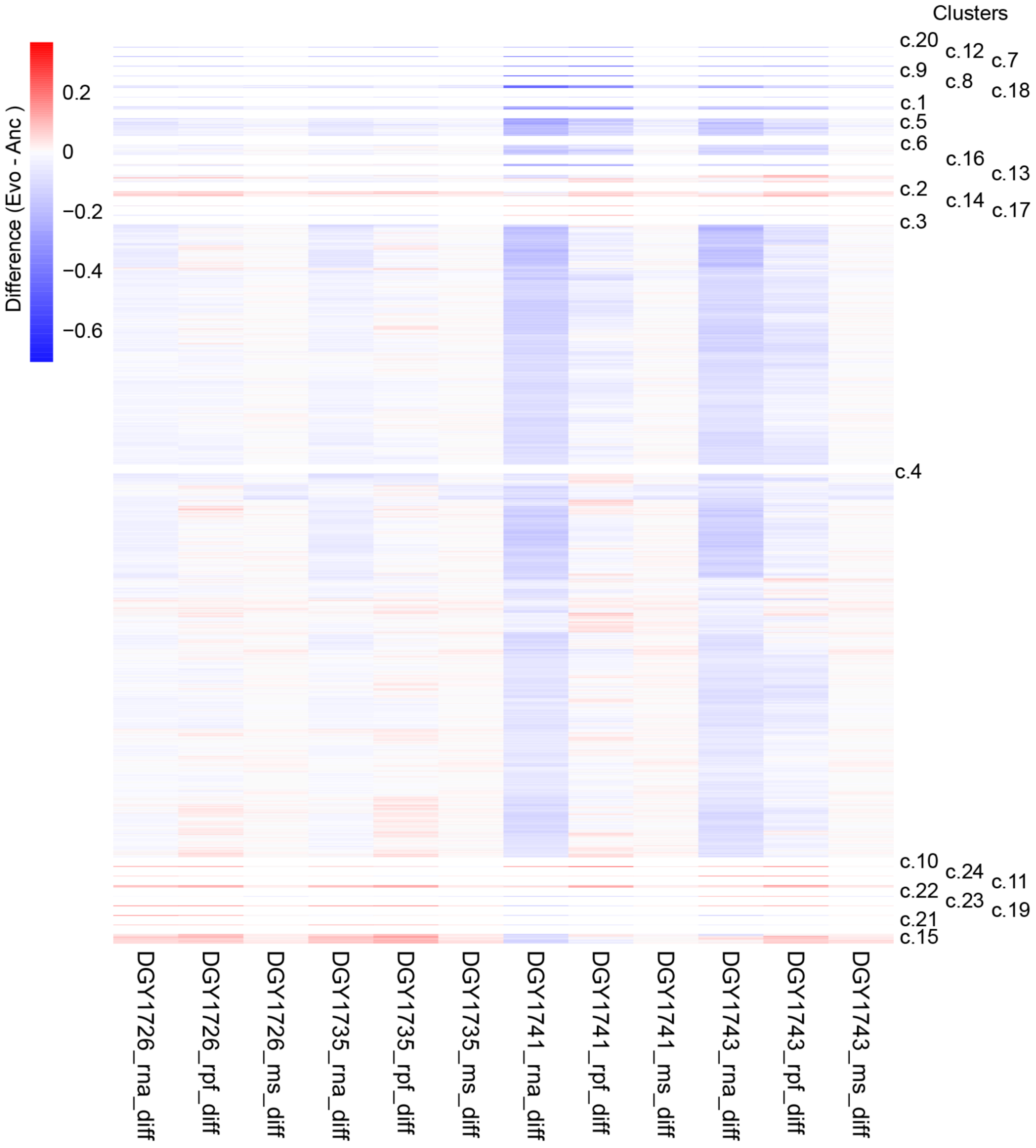

Figure S9: Heatmap of multi-level expression with clustering (k=24). Heatmap shows the difference (Evolved - Ancestor) of the Unit Transformed expression data for mRNA, RPF, and MS intensity for all evolved strains. Cluster assignment is available in **Table ST7**.

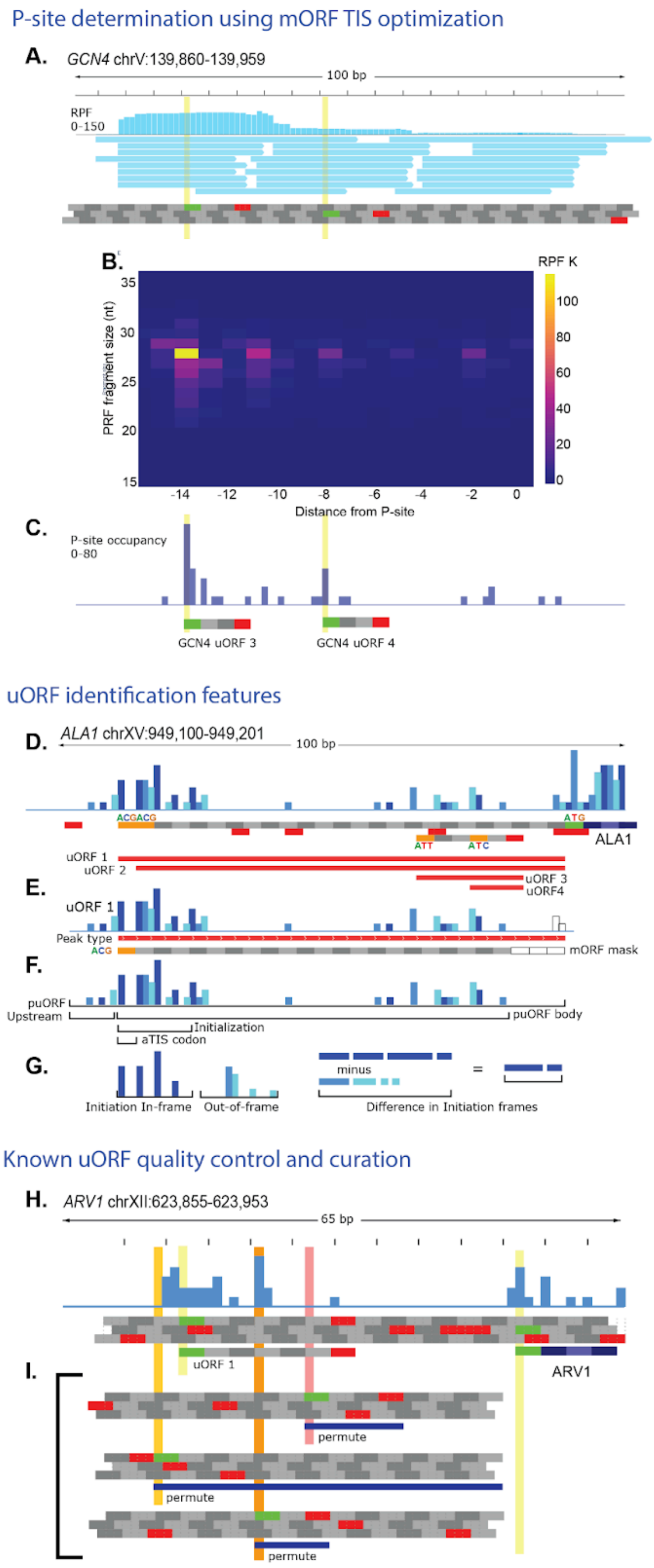
P-site determination using mORF TIS optimization
A.
GCN4 chrV:139,860-139,959
100 bp
RPF
0-150
B.
RPF K
PRF fragment size (nt)
Distance from P-site
C.
P-site occupancy
0-80
GCN4 uORF 3
GCN4 uORF 4
uORF identification features
D.
ALA1 chrXV:949,100-949,201
100 bp
ACGACG
ATG
ATT
ATC
ALA1
uORF 1
uORF 2
uORF 3
uORF4
E.
uORF 1
Peak type
ACG
mORF mask
F.
puORF
Upstream
puORF body
Initialization
aTIS codon
G.
minus
=
Initiation In-frame
Out-of-frame
Difference in Initiation frames
Known uORF quality control and curation
H.
ARV1 chrXII:623,855-623,953
65 bp
uORF 1
ARV1
I.
permute
permute
permute

Figure S10. uORF identification and scoring workflow diagram. For each ribosome profiling sample uORFish performs the following steps before training a model: step 1) p-site determination, step 2) known uORF curation, and step 3) feature scoring.

The accurate assignment of ribosomes to reading frames is essential for the identification of uORFs (**A**). To address this we find the optimal offset for each fragment size to maximize the p-site occupancy using the annotated start codons of protein coding regions (**B**). This fragment size specific offset is then used to transform RPFs into p-site abundances (**C**).

Unlike previous ML approaches (Spealman et al. 2021) that required both ribosome profiling and RNAseq data in triplicate, we sought to use features derived from only a single replicate of ribosome profiling data. An example of these features are shown using the alternative translation initiation sites (aTISs) of ALA1 (YOR335C). ALA1 encodes an alanyl-tRNA synthetase that has two proteoforms, the one that uses the canonical “AUG” start codon is localized to the cytoplasm, while the longer version using one of two “ACG” start codons is localized to the mitochondria (Chang et al. 2010) (**D**).

Scored features include two categories; start codon and peak/ramp distribution. These categories are used to compare potential uORFs (puORFs) to relevant peers. For example, the scored features for an ACG type uORF should not be expected to have the same distribution as AUG type uORFs. Similarly, while some uORFSs have well defined “peaks” of p-site abundances on their TIS (such as those shown for uORF 1 and 2, **D**) others exhibit “ramps” (such as uORF 1 of ARV1, **H**), potentially indicative of ribosome queueing. As such puORFs are categorized both by start codon sequence and p-site distribution type. Each puORF is also scored using regional features (**F**) such as total occupancy upstream, over the body, over the initialization region (Verma et al. 2019) and at the aTIS itself. Finally, we calculate in-frame and out-of-frame scores for each of these regions as well as composites scores, such as the difference in abundance of in-frame and out-of-frame p-sites (**E**).

Finally we want to train our model using feature performance scored on molecularly validated uORFs. However, these uORFs have been validated by a variety methods and under a variety of environmental conditions (Hinnebusch 2005; Spealman and Naik et al. 2018; Eisenberg et al. 2020; May et al. 2023) that, potentially, are not relevant to the experimental condition under consideration. As such, we implement a curation step that compares the performance of each validated uORF against a random null model. An example of this is shown using gene ARV1 (YLR242C), validated by May et al. 2023 (**H**). Each categorical combination of start codon and peak distribution type has its own null model. Each null model is generated by first randomly selecting a gene, randomly scrambling the triplet nucleotide sequence of the TL, then scanning and scoring any uORFs generated at random, this is performed a minimum of 10,000 times per model (**I**). To be considered for training a puORF must have a score that is equal to, or exceeds, the top 5% of the null model scores. In this example ARV1 scores at a 7.8% so is not used for training the model.

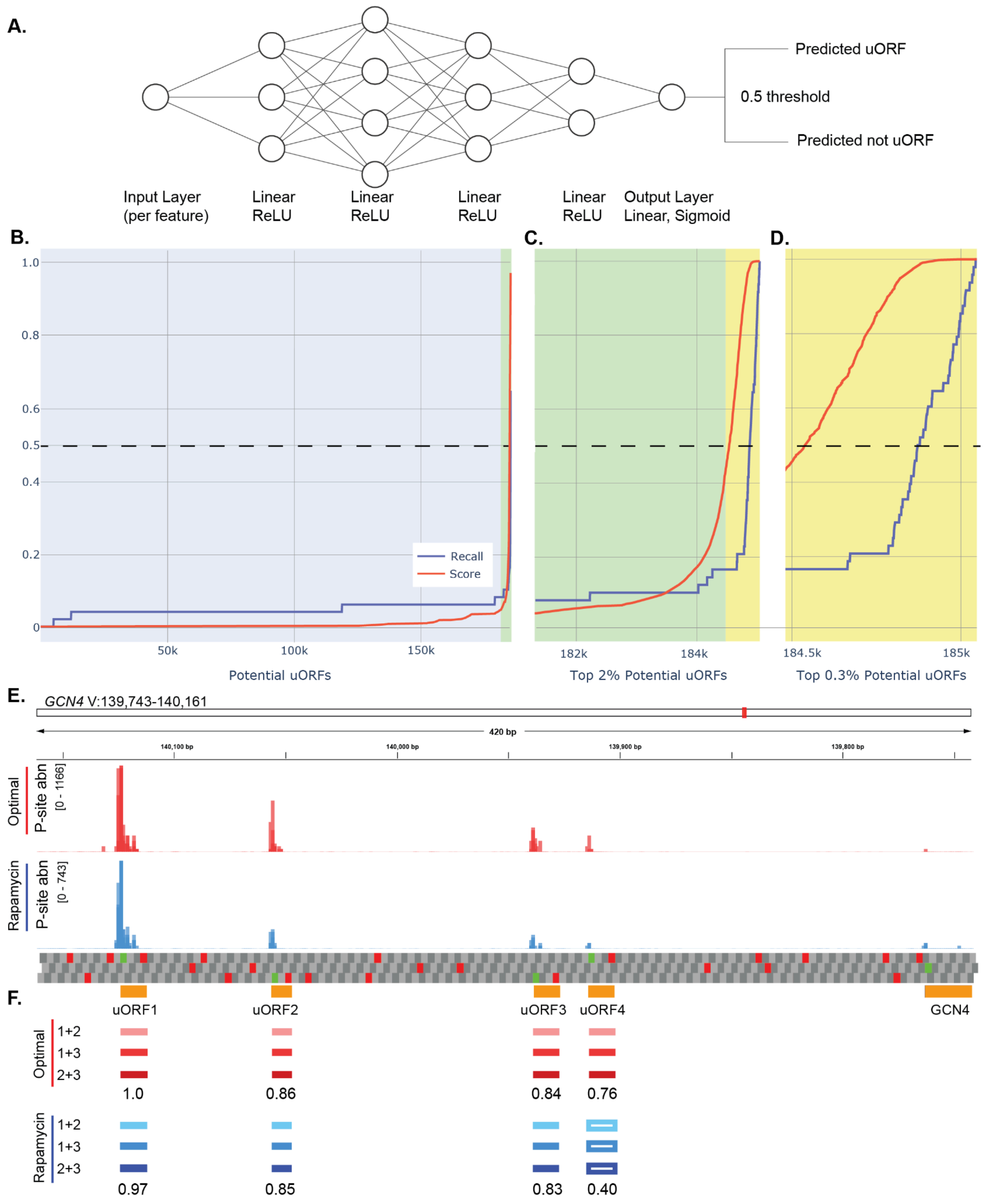

Figure S11. uORF identification and scoring workflow diagram. Network diagram for the DNN showing the layers for each feature (**A**), note the sigmoid output layer has a classification threshold of 0.5. One evaluation of DNN performance comparing Score (red line) and Recall

(blue line, percent True, eg. molecularly validated uORFs). Three ranges are shown for performance, including the whole range (**B**), the top 2% (**C**) and the top 0.3% (**D**). With a classification threshold of 0.5 (dotted line) we see that only the top performing ~ 0.25% of potential uORFs are classified as uORFs, with more than 80% of the molecularly validated uORFs being correctly classified as such.

The long right hand Recall tail is composed of molecularly validated uORFs that are often underutilized in the evaluated condition, one example of this is *GCN4* uORF 4 (**F**). Here we show P-site abundances for both Optimal and Rapamycin conditions (Nedialkova and Leidel 2015), we also show the respective resolved predictions made by *uORFish*. Resolved uORFs are the highest scoring, non-overlapping uORFs present in two replicates, and each row shows the agreement between two replicates (1 and 2, 1 and 3, 2 and 3). Mean scores across combined replicates are shown. This shows a high degree of agreement and suggests that the use of only two replicates, as is the case in the main study, is well-powered. Notably, we find uORF 4 fails to be called in the Rapamycin treatment condition (blue hollow boxes, mean score = 0.40). This is in agreement with uORF 4 being most active under optimal growth conditions, whereas the translation of uORF 4 prevents the translation of *GCN4* (Abastado et al. 1991).

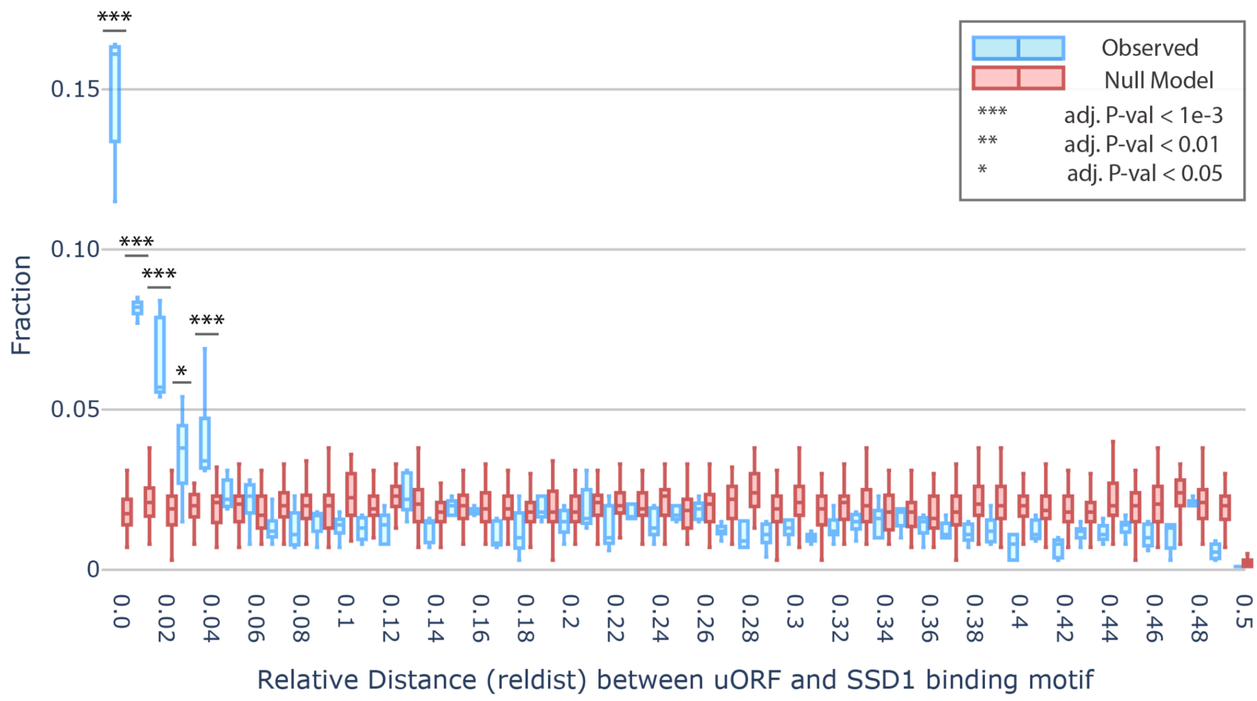

Figure S12: Relative distance between uORF and SSD1 binding motif: Boxplot showing the relative distance (Favorov et al. 2012), between overlapping uORFs and SSD1 motifs in transcript leaders (0 - 0.5). Vertical axis shows the fraction of total co-occurrences observed at each percentage distance (median, all strains). The co-occurence of uORFs and SSD1 binding motifs observed (blue) versus a null model (red). The null model is the median of ten randomly shuffled genome sequences, SSD1 motifs were identified in the shuffled genome, and the distances from these to the identified uORFs was calculated for each strain. Reldist was calculated using bedtool's *reldist* function (Quinlan and Hall 2010). The first five percent positions (0.0 - 0.04) have significantly higher rates of co-occurrence than the null model, suggesting that there is close local positional enrichment of these two regions.

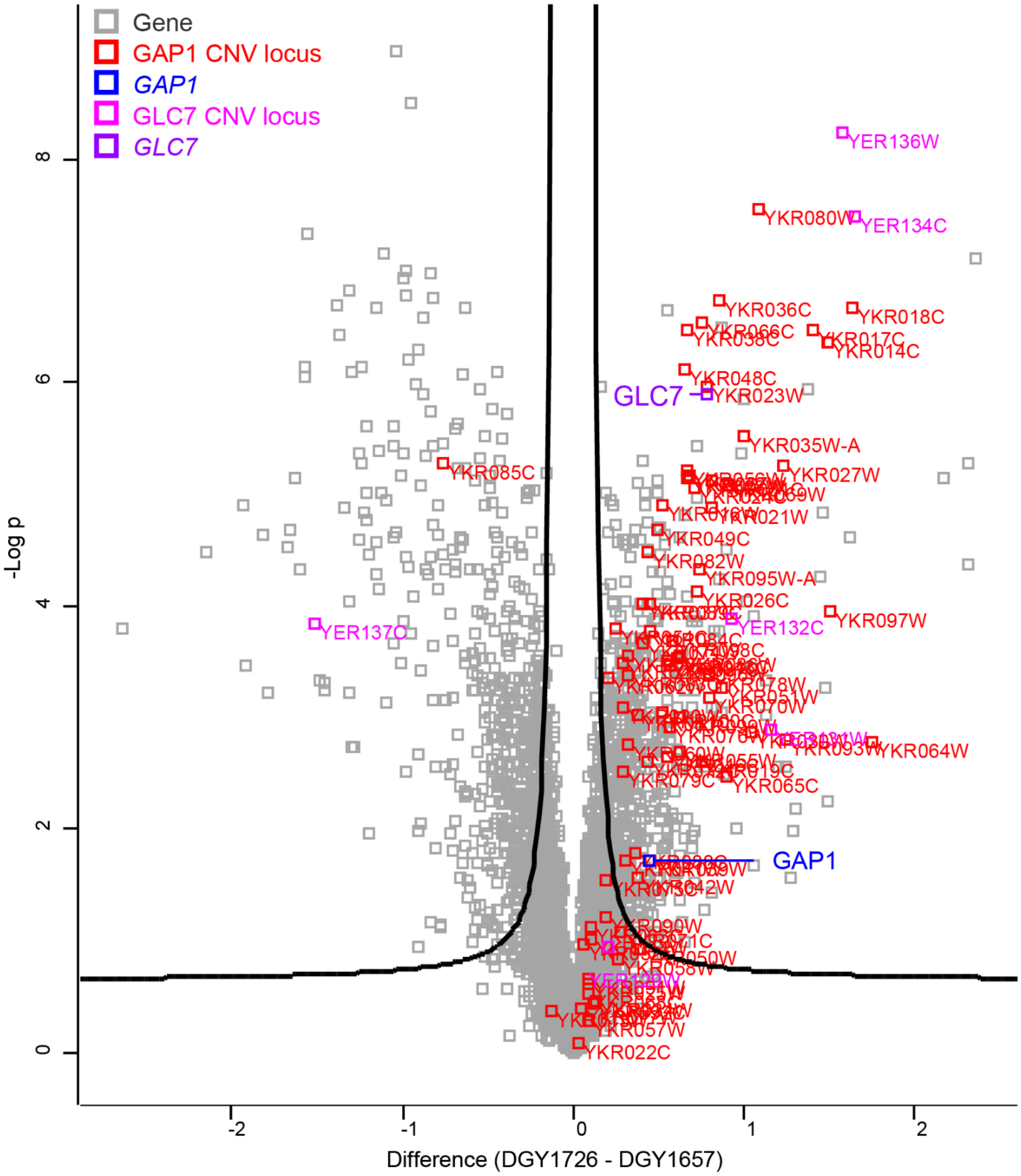

Figure S13: Differential Protein Abundance Results for Trans (DGY1726). Volcano plot of results of Welch's t-test between Trans (DGY1726) and Ancestor (DGY1657). Two regions of the genome have been CNV amplified. Genes amplified with the GAP1 locus are shown in red, *GAP1* is shown in blue. Genes amplified with the GLC7 locus are shown in pink, *GLC7* is shown in purple.

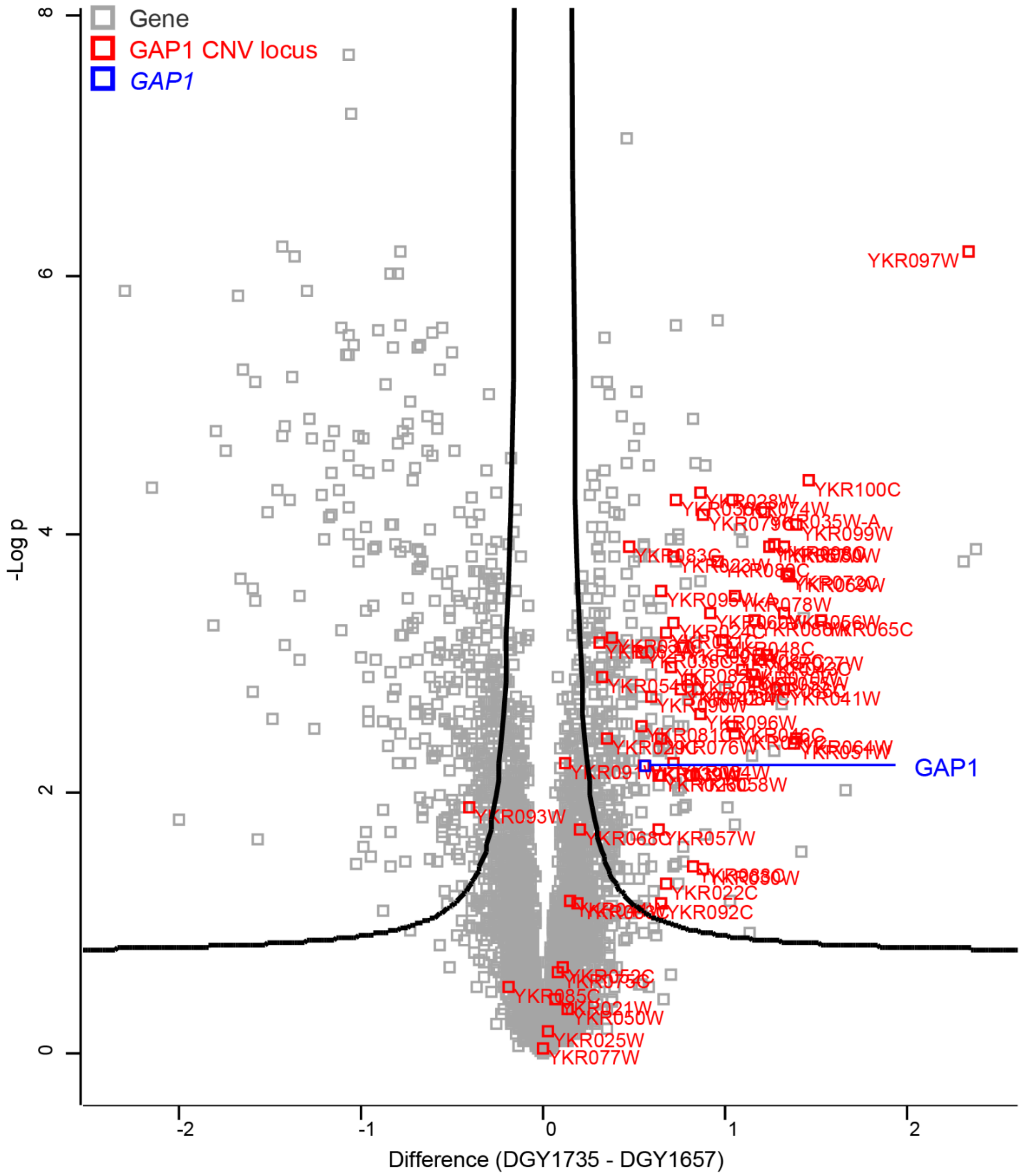

Figure S14: Differential Protein Abundance Results for ODIRA_A (DGY1735). Volcano plot of results of Welch's t-test between ODIRA_A (DGY1735) and Ancestor (DGY1657). Genes amplified in the GAP1 locus are shown in red, *GAP1* is shown in blue.

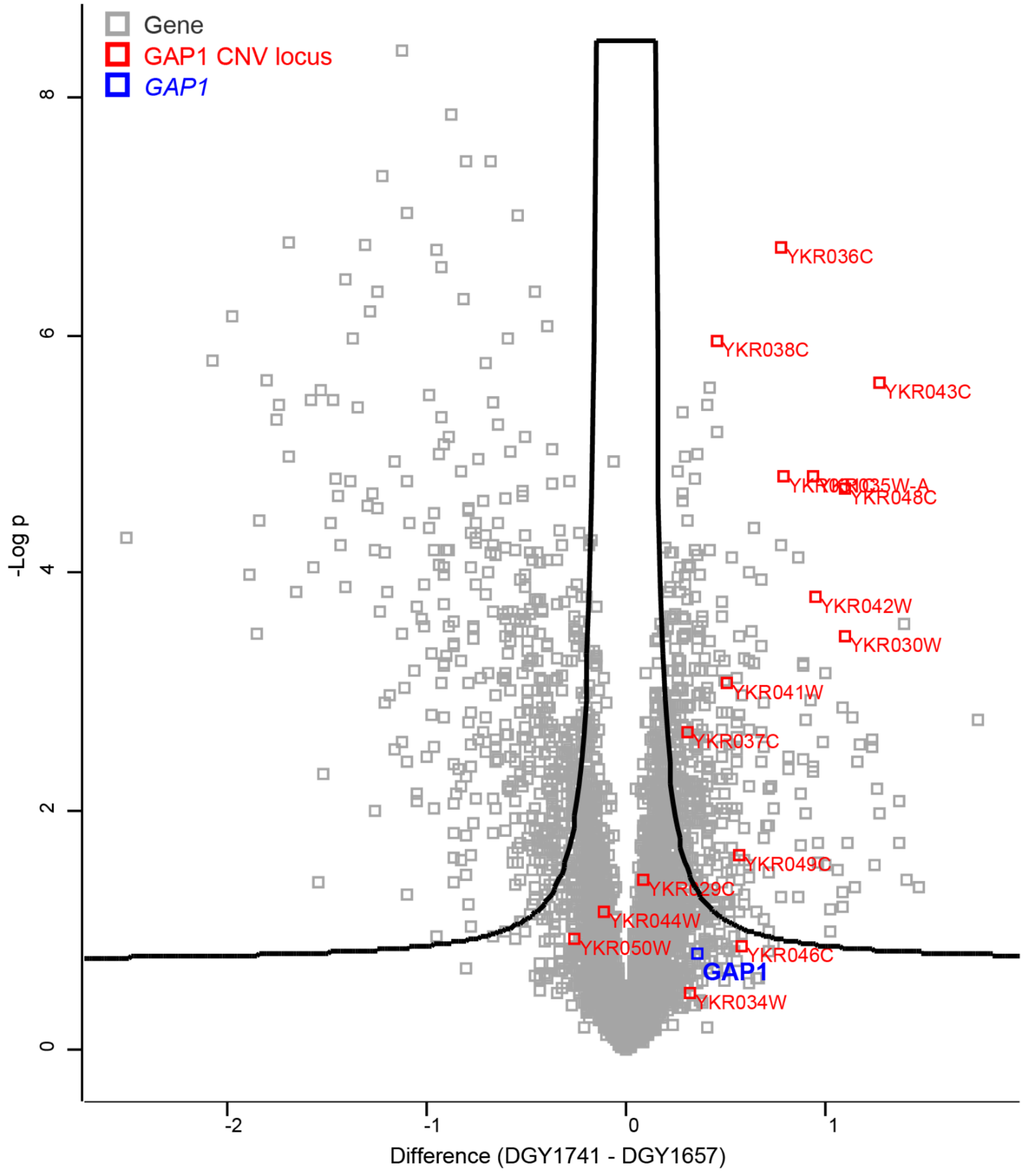

Figure S15: Differential Protein Abundance Results for ComQuad (DGY1741). Volcano plot of results of Welch's t-test between ComQuad (DGY1741) and Ancestor (DGY1657). Genes amplified in the GAP1 locus are shown in red, *GAP1* is shown in blue. Note that *GAP1* protein abundance in ComQuad is not significantly different from the Ancestor despite having been amplified to a copy-number of 4.

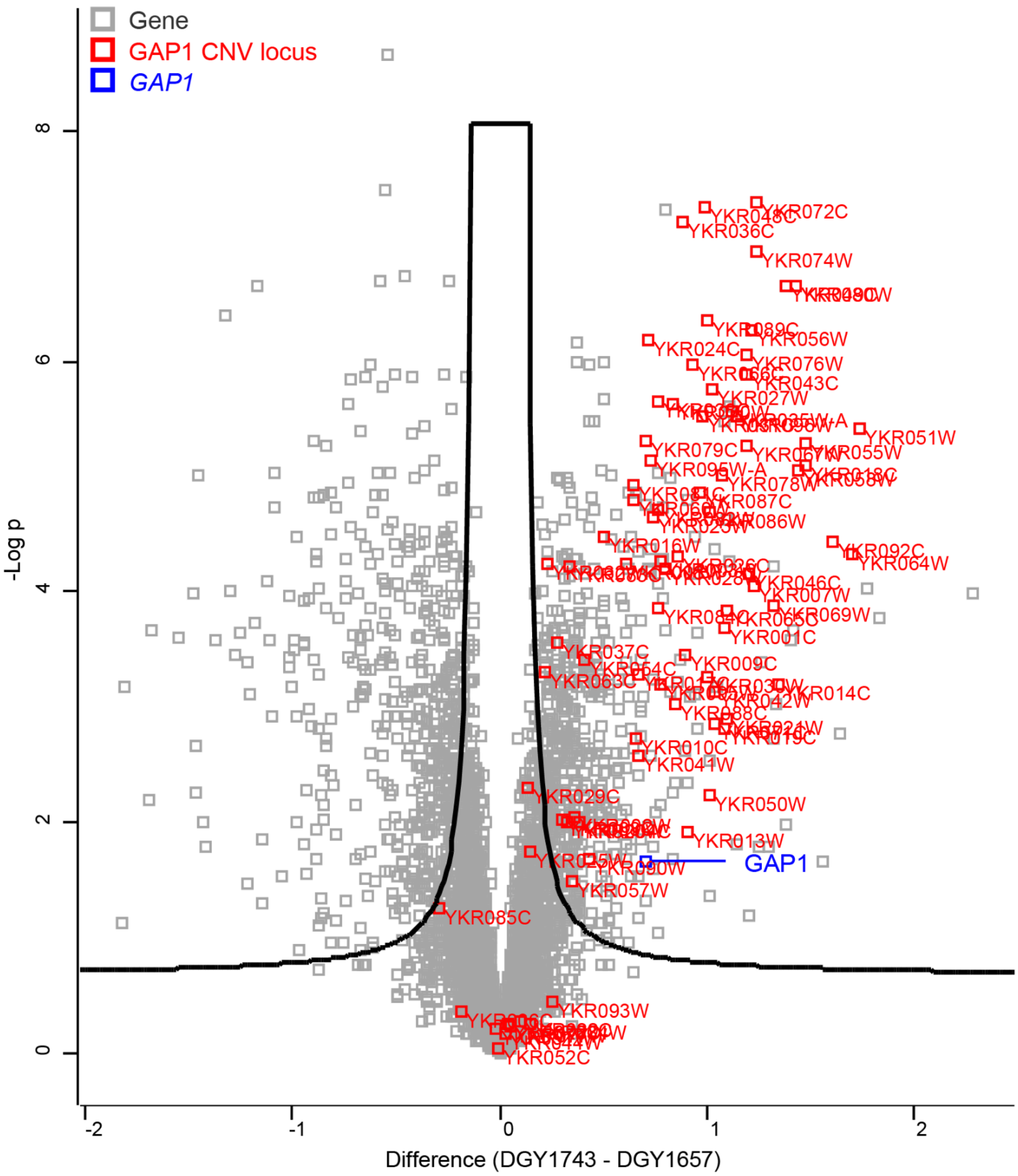

Figure S16: Differential Protein Abundance Results for ODIRA_B (DGY1743). Volcano plot of results of Welch's t-test between ODIRA_B (DGY1743) and Ancestor (DGY1657). Genes amplified in the GAP1 locus are shown in red, *GAP1* is shown in blue.

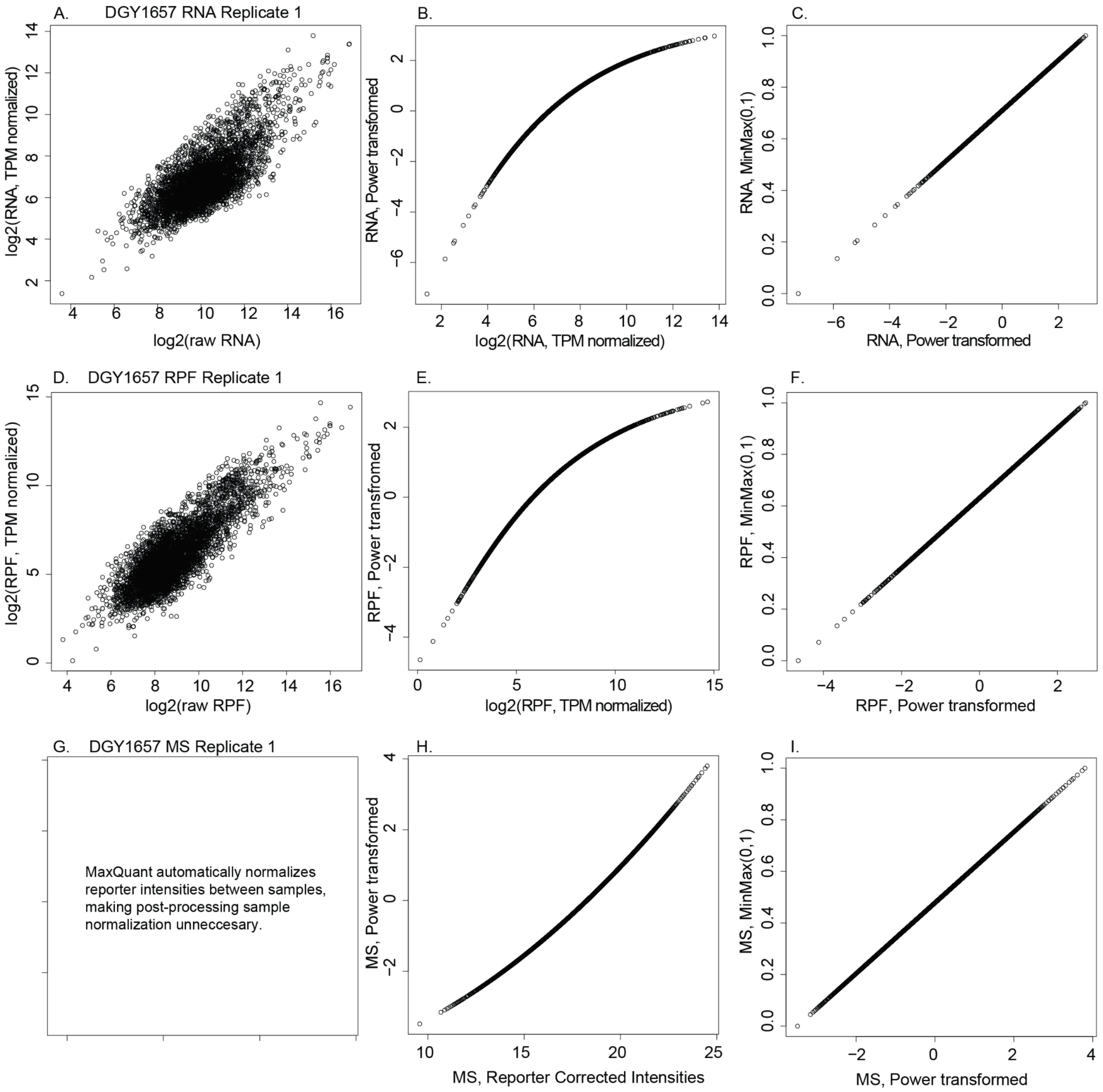

Figure S17: Example of Power Transform of Expression Data. In order to evaluate changes in protein efficiency (MS/RPF) we wanted to have a robust differential test that could evaluate these two levels of expression. One critical step is transforming and scaling the data. Large numerical differences in scale separate expression data, with RNA and RPF ranging from 10-100,000 read counts per gene compared to 10-25 peptide reporter intensities from MS data. To compare these directly we sought to transform the data into similarly scaled units. For RNA and RPF data (**A, D**) we first normalize between samples for sequencing depth using TPM (Li and Dewey 2011). Next (**B, E, H**), we bring measurements within a similar scale using a Box-Cox Power Transform (Box and Cox 1964). This has the added benefit of retaining, reinforcing the Gaussian distribution. This is followed by a MinMax transformation (**C, F, I**) to keep all values positive. Both Power Transform and MinMax were implemented using Scikit-learn (Pedregosa et al. 2011).

A. General Linear Model for Translation Efficiency

```
RPF, RNA data has not been transformed

Evo_rpf_median = median(Evo RPF_rep_1, Evo RPF_rep_2)
Evo_rna_median = median(Evo RNA_rep_1, Evo RNA_rep_2)
Anc_rpf_median = median(Anc RPF_rep_1, Anc RPF_rep_2)
Anc_rna_median = median(Anc RNA_rep_1, Anc RNA_rep_2)

glm_fit = glm(
(Evo_rpf_median) ~ (Anc_rna_median * Anc_rpf_median * Evo_rna_median),
        family = gaussian)
```

B.

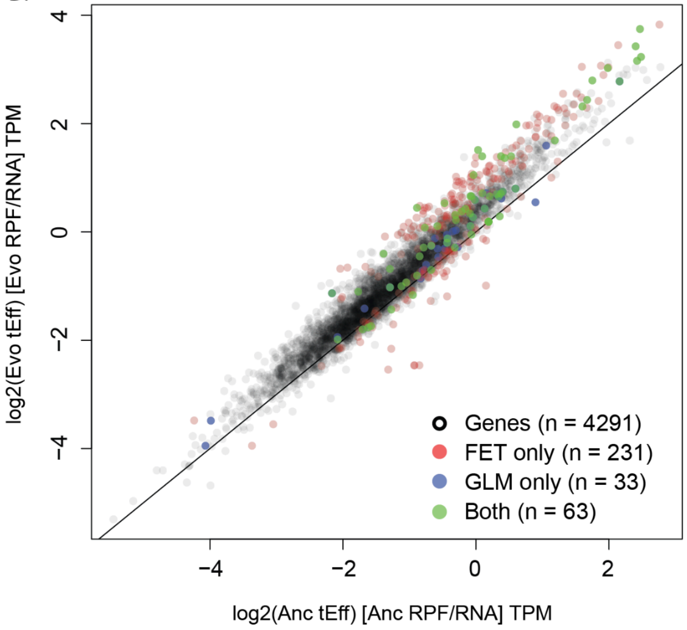

C.

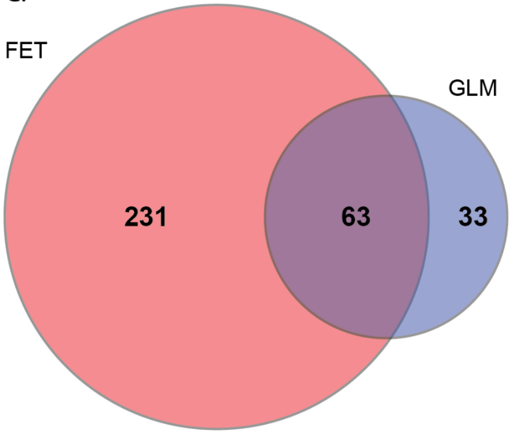

F1	0.323
FPR	0.008
FDR	0.344
Accuracy	0.938
Sensitivity	0.214

Figure S18: Generalized linear model performance on translation efficiency data. To identify changes in efficiency without transforming the data we used a Gaussian generalized linear model, here written for translation efficiency using RPF and RNA data from the Trans (DGY1726) strain (**A**). Genes with standardized residuals greater than 1.96 were defined as significant outliers. The results of this model were compared to the results of an FET analysis of the same data (**B**). FET identified 294 genes with significantly different translation efficiency, GLM identified 96 genes, of which 63 genes were identified by both methods (**C**). We can compare results between methods by letting the FET results represent the ideal identification case, such that if a gene is identified by both FET and GLM it constitutes a true positive (TP), if absent from GLM a false negative (FN), absent from FET a false positive (FP), and absent from both a true negative (TN). F1 score = 2TP / (2TP + FP + FN); FPR = FP / (FP + TN); FDR = FP / (FP + TP); Accuracy = (TP + TN) / (Total Positive + Total Negative); Sensitivity = TP / (TP + FN).

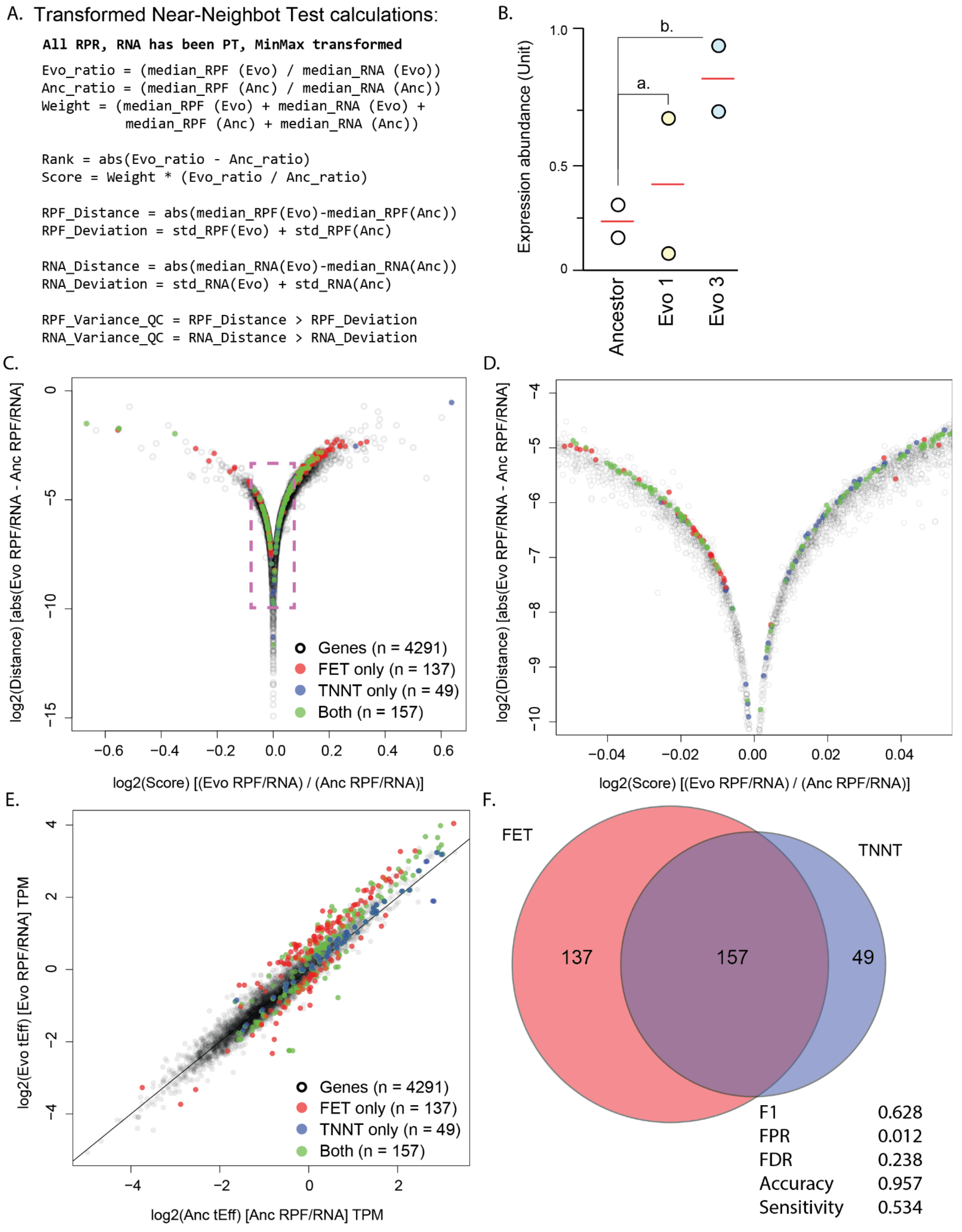

Figure S19: Example of TNNT at two levels of expression. We also evaluated the performance of a transformed data and near-neighbor test (TNNT) using translation efficiency data. After scaling the expression data each gene is **ranked** using the distance between the protein efficiency ratios and **scored** using the ratio multiplied by the weight (**A**). Genes with in-sample variance greater than difference are removed (eg. gene “a” in **B**). The FDR is determined by subsampling. Similar to the GLM analysis, we can compare the performance to FET. Using a distance by score plot we find close agreement globally (**C**) and locally (**D**). Similarly after mapping these results back into untransformed units (**E**). FET identified 294 genes, TNNT identified 206 genes, 157 of which were also identified by FET (**F**). Comparison metrics are calculated as above (Figure S1).

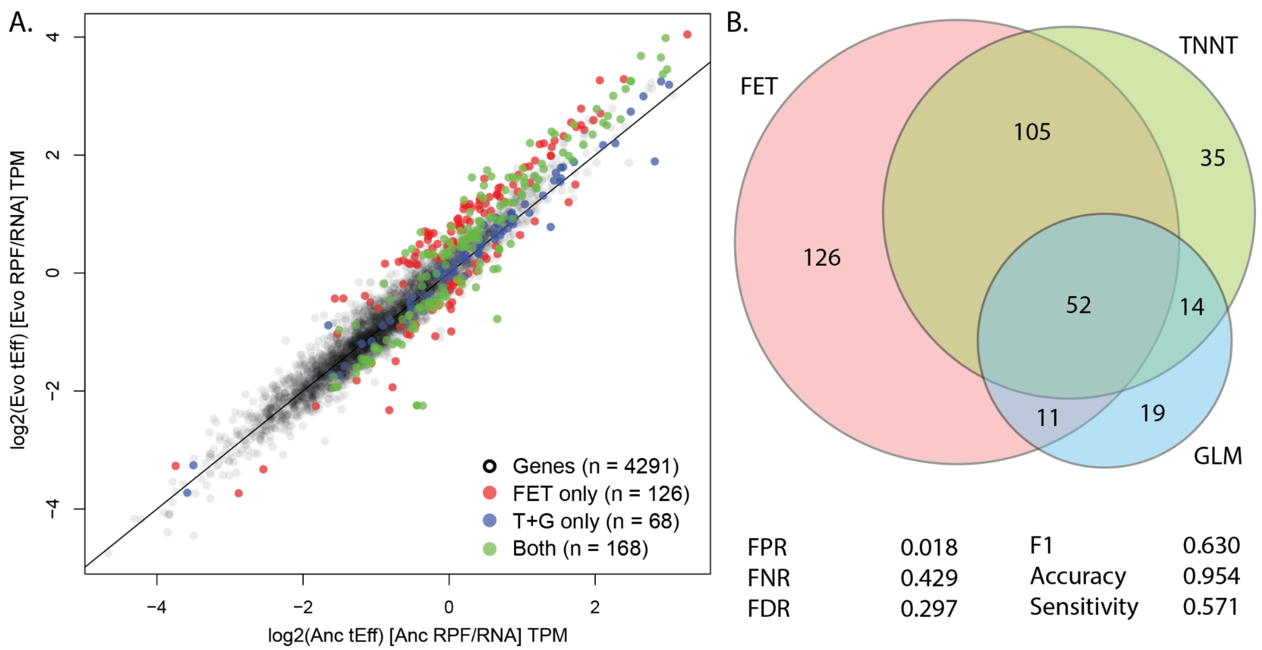

Figure S20: Combined analysis of translation efficiency using both TNNT and GLM. We also sought to compare the performance between the TNNT and GLM methods. Projecting the identified genes into untransformed evolved and ancestor efficiencies we find broad agreement (**A**) with 11 additional novel identifications made by the GLM method. Performance compared to the FET test of translation efficiency ratios finds reasonable agreement with a false positive rate of 0.02, a false negative rate of 0.43 and a F1 score of 0.63 (**B**). This additive combination of GLM and TNNT methods (now referred to as **TNNTg**) is used for further analysis or protein expression efficiency data (Figure 4 in the main text).

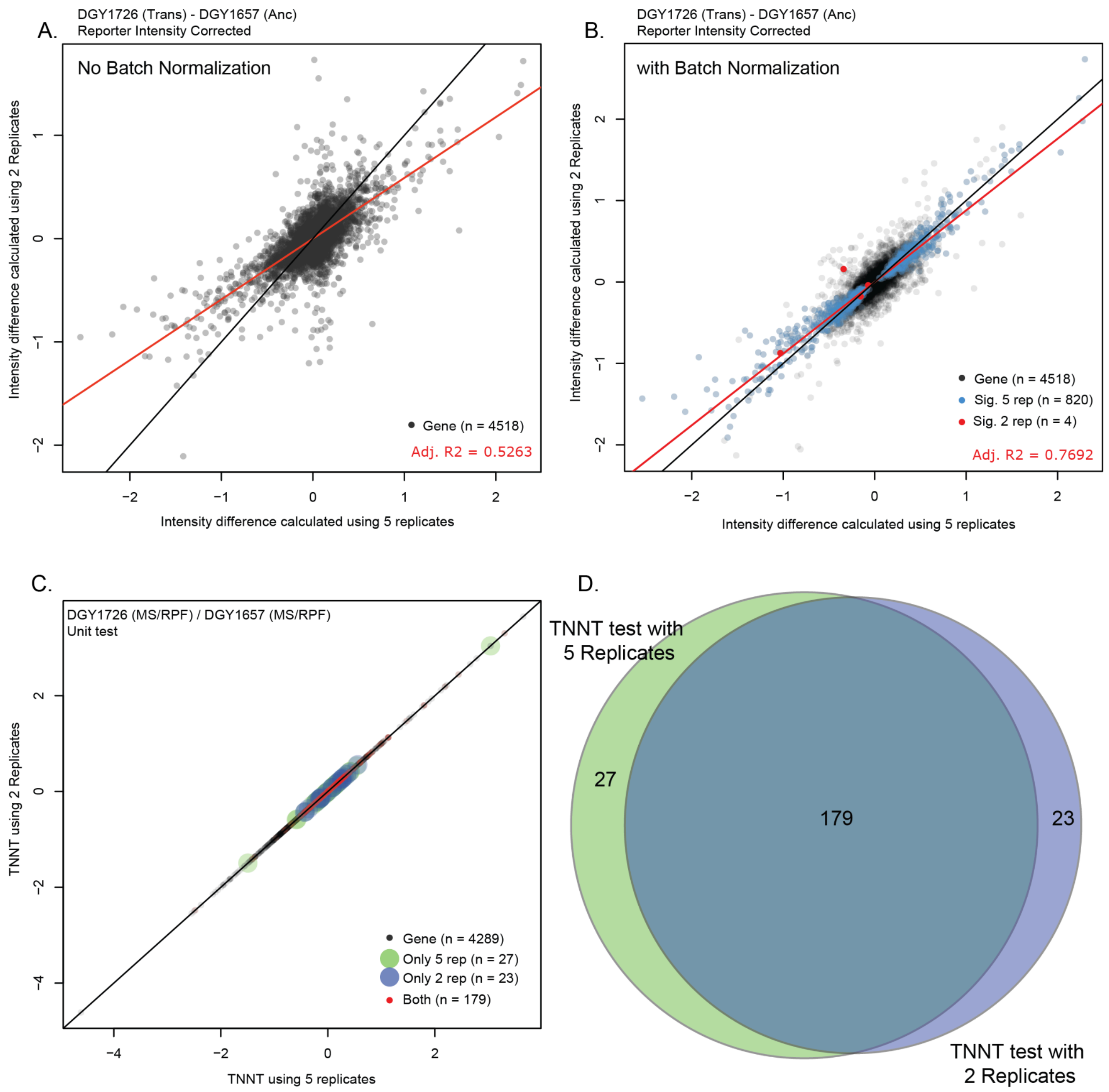

Figure S21: Effect of imbalance in replicates on analysis and TNNT performance. For each strain we have 2 replicates of RNAseq and ribosome profiling and 5 replicates of mass spectrometry. While variance in MS measurements usually requires more replicates than NGS data, we sought to evaluate the effect this imbalance has on our analysis. To do so we re-ran our proteomics analysis for strain DGY1726 using only 2 MS replicates (replicates 1 and 2). Batch normalization is a critical step in MS experiments (Yu, Kyriakidou, and Cox 2020), which relies on higher numbers of replicates (3+). Removing this step does remove a significant component of the signal as the background variance is unreduced. (**A**) We performed the Welch's T-test using data without batch normalization for both the 2 and 5 replicate data and found low correlation between the two analyses (adj.R^2 = 0.51).

We next kept all MS replicates to perform batch normalization before removing them for all further downstream analysis. (**B**) Re-running Welch's t-test with batch correction we find a much higher

correlation (adj.$R^2 = 0.77$) - however it is still very statistically underpowered with only 4 genes identified as significantly different (BH. adj. pval < 0.05). (**C**) Finally, we can evaluate the effect this has on protein efficiency by using the TNNT. Using this approach we find a very high correlation between the MS/RPF values of the 5 replicate and 2 replicate runs (adj $R^2 \sim 1$), furthermore gene agreement is also highly similar (**D**).

Supplemental tables:

Supplemental table 1: ST1_chemostat_gene_relative_copy_number.tsv

Table containing resolved copy number on a gene by gene by strain basis.

Supplementary Table ST2: Spearman *rho* correlation between replicates and between levels of expression.

**DGY1657**	RNA	RPF	MS
RNA	0.98966	0.70425	0.584004
RPF		0.990533	0.628283
MS			0.996504

**DGY1726**	RNA	RPF	MS
RNA	0.987811	0.635839	0.573157
RPF		0.959884	0.60123
MS			0.998446

**DGY1735**	RNA	RPF	MS
RNA	0.987192	0.627072	0.545272
RPF		0.979158	0.61208
MS			0.99717

**DGY1741**	RNA	RPF	MS
RNA	0.991494	0.612493	0.508551
RPF		0.992748	0.582381
MS			0.989193

**DGY1743**	RNA	RPF	MS
RNA	0.982327	0.618318	0.50822
RPF		0.987891	0.595538
MS			0.993212

Supplementary Table ST3: Spearman *rho* correlation between strains and between levels of expression.

**RNA**	DGY1726	DGY1735	DGY1741	DGY1743
DGY1657	0.963762	0.945636	0.885821	0.906666
DGY1726		0.978994	0.912389	0.939575
DGY1735			0.904653	0.945145
DGY1741				0.939695

**RPF**	DGY1726	DGY1735	DGY1741	DGY1743
DGY1657	0.946509	0.946274	0.892832	0.922275
DGY1726		0.9527	0.892116	0.939025
DGY1735			0.876115	0.929458
DGY1741				0.916981

**MS**	DGY1726	DGY1735	DGY1741	DGY1743
DGY1657	0.990968	0.990221	0.987052	0.989726
DGY1726		0.996515	0.991433	0.996051
DGY1735			0.991952	0.996022
DGY1741				0.991495

Supplemental Table ST4: Results of DESeq2 on RNA

Table containing the results of DESeq2 pairwise tests performed between each evolved strain and the ancestor using observed RNA abundances.

Supplemental Table ST5: Results of DESeq2 on RPF

Table containing the results of DESeq2 pairwise tests performed between each evolved strain and the ancestor using RPF abundances.

Supplemental Table ST6: Results of Welch's t-test on MS intensities

Table containing the results of Welch's t-test pairwise tests performed between each evolved strain and the ancestor using mass spectrometry instensities.

Supplemental Table ST7: Genes with cluster assignment

Table containing the gene name and assigned cluster for all genes in Figure 2A and Supplemental Figure 9.

Supplemental Table ST8: Results of DESeq2 on RNA Expected versus Observed

Table containing the results of DESeq2 pairwise tests performed between the expected RNA abundance of each evolved strain and the observed RNA abundance in the ancestor.

Supplemental Table ST9: Summary of Genes with Increased Transcription Efficiency

**SigUp**	**Sig CNN**	**CNN**	**pct**	**Sig CNV**	**CNV**	**pct**	**FET, pval**
**DGY1726**	93	4207	2.2	3	100	3	0.49
**DGY1735**	167	4222	4	4	77	5.2	0.55
**DGY1741**	909	4274	21.3	5	18	27.8	0.58
**DGY1743**	569	4210	13.5	20	91	22	0.64
**SigDown**	**Sig CNN**	**CNN**	**pct**	**Sig CNV**	**CNV**	**pct**	**FET, pval**
**DGY1726**	257	4207	6.1	9	84	10.7	0.12
**DGY1735**	440	4222	10.4	11	69	15.9	0.18
**DGY1741**	1112	4274	26	4	17	23.5	1
**DGY1743**	916	4210	21.8	19	81	23.5	0.79

Table containing the number of genes per strain that have significantly higher (Up, first 4 rows) or lower (Down, last four rows) transcription efficiency (DESeq2, adj.pval <= 0.05). No significant difference is found between CNVs and copy-number normal (CNN) genes.

Supplemental Table ST10: Results of Fisher's Exact Test on Translation Efficiency

Table containing the results of Fisher's exact test (FET) conducted pairwise on ratios of TPM transformed RPF and TPM transformed RNA abundances for evolved strain and the ancestor.

Supplemental Table ST11: Results of TNNTg on MS and RPF

Table containing the results of TNNTg. The test was conducted pairwise on ratios of unit transformed MS and unit transformed RPF abundances for evolved strain and the ancestor.

Supplemental Table ST12: GATK Variant Calls

Single nucleotide variants and indels identified by GATK in the strains in conjunction with variant effect prediction annotation by Ensembl VEP when possible. GATK was run using default options against a modified reference genome containing the Gresham GFP reporter (Lauer et al. 2018). For Supplemental Figure 2 these were further reduced for the evolved by removing variants present in the ancestor.

Supplemental Table ST13: CVish predicted boundaries

CNV/SV boundaries for all strains predicted by CVish using Illumina sequencing reads. CVish was run using default options against a modified reference genome containing the Gresham GFP reporter (Lauer et al. 2018). Each tab represents a strain and uses the gff format.

Supplemental Table ST14: uORFish predicted uORF results.

Table containing the results of *uORFish* (v1.3) run (using default) on the ribosome profiling data for each replicate of each strain. uORFish was run using default options, all potential predictions with a score of at least 0.5 are included. Only uORFs with scores of at least 0.5 in both replicates were included in the study.

Supplemental Table ST15: Results of SSD1 motif scan

Table containing the results of a motif search for the conserved *SSD1* motif ('CNYUCNYU', reported by Bayne et al. 2022) located within the transcript leaders (5'UTRs) of protein coding genes.

Supplemental files:

Supplemental File 1: SF1_Saccharomyces_cerevisiae.R64-1-1.ncrna_wo_ncrna_genes.fa

Custom fasta file used to filter out snRNA, snoRNA, rRNA, and tRNA but not potentially translated non-coding RNA.

Supplemental File 2: SF2_MaxQuant_16plex_template_10262023.txt

Isobaric weight template for MaxQuant for 16plex TMT-labeled MS.

Supplemental File 3: SF3_parameters.txt

Parameters file for MaxQuant analysis for MS data.

Works cited:

Abastado, J. P., P. F. Miller, B. M. Jackson, and A. G. Hinnebusch. 1991. “Suppression of Ribosomal Reinitiation at Upstream Open Reading Frames in Amino Acid-Starved Cells Forms the Basis for GCN4 Translational Control.” *Molecular and Cellular Biology* 11 (1): 486–96.

Box, G. E. P., & Cox, D. R. (1964). An Analysis of Transformations. *Journal of the Royal Statistical Society. Series B (Methodological)*, *26*(2), 211–252. http://www.jstor.org/stable/2984418

Favorov, Alexander, Loris Mularoni, Leslie M. Cope, Yulia Medvedeva, Andrey A. Mironov, Vsevolod J. Makeev, and Sarah J. Wheelan. 2012. “Exploring Massive, Genome Scale Datasets with the GenometriCorr Package.” *PLoS Computational Biology* 8 (5): e1002529.

“GitHub - Pspealman/heranca: A Small Script for Reducing False Positive Variant Calls (VCFs) Using Lineage Information.” n.d. GitHub. Accessed October 24, 2023. https://github.com/pspealman/heranca.

Li, Bo, and Colin N. Dewey. 2011. “RSEM: Accurate Transcript Quantification from RNA-Seq Data with or without a Reference Genome.” *BMC Bioinformatics* 12 (August):323.

McLaren, William, Laurent Gil, Sarah E. Hunt, Harpreet Singh Riat, Graham R. S. Ritchie, Anja Thormann, Paul Flicek, and Fiona Cunningham. 2016. “The Ensembl Variant Effect Predictor.” *Genome Biology* 17 (1): 122.

Nedialkova, Danny D., and Sebastian A. Leidel. 2015. “Optimization of Codon Translation Rates via tRNA Modifications Maintains Proteome Integrity.” *Cell* 161 (7): 1606–18.

Pedregosa, Fabian, Gaël Varoquaux, Alexandre Gramfort, Vincent Michel, Bertrand Thirion, Olivier Grisel, Mathieu Blondel, et al. 2011. “Scikit-Learn: Machine Learning in Python.” *Journal of Machine Learning Research: JMLR* 12 (85): 2825–30.

Quinlan, Aaron R., and Ira M. Hall. 2010. “BEDTools: A Flexible Suite of Utilities for Comparing Genomic Features.” *Bioinformatics* 26 (6): 841–42.

Robinson, James T., Helga Thorvaldsdóttir, Wendy Winckler, Mitchell Guttman, Eric S. Lander, Gad Getz, and Jill P. Mesirov. 2011. “Integrative Genomics Viewer.” *Nature Biotechnology* 29 (1): 24–26.

Van der Auwera, Geraldine A., Mauricio O. Carneiro, Christopher Hartl, Ryan Poplin, Guillermo Del Angel, Ami Levy-Moonshine, Tadeusz Jordan, et al. 2013. “From FastQ Data to High Confidence Variant Calls: The Genome Analysis Toolkit Best Practices Pipeline.” *Current Protocols in Bioinformatics / Editoral Board, Andreas D. Baxevanis ... [et Al.]* 43 (1110): 11.10.1–11.10.33.

Verma, Manasvi, Junhong Choi, Kyle A. Cottrell, Zeno Lavagnino, Erica N. Thomas, Slavica Pavlovic-Djuranovic, Pawel Szczesny, et al. 2019. “A Short Translational Ramp Determines the Efficiency of Protein Synthesis.” *Nature Communications* 10 (1): 5774.

Yu, Sung-Huan, Pelagia Kyriakidou, and Jürgen Cox. 2020. “Isobaric Matching between Runs and Novel PSM-Level Normalization in MaxQuant Strongly Improve Reporter Ion-Based Quantification.” *Journal of Proteome Research* 19 (10): 3945–54.
